# Chromism-Integrated Sensors and Devices for Visual Indicators

**DOI:** 10.3390/s22114288

**Published:** 2022-06-04

**Authors:** Hyunho Seok, Sihoon Son, Jinill Cho, Sanghwan Choi, Kihong Park, Changmin Kim, Nari Jeon, Taesung Kim, Hyeong-U Kim

**Affiliations:** 1 SKKU Advanced Institute of Nanotechnology (SAINT), Sungkyunkwan University, Suwon 16419, Korea; dddsjk@skku.edu (H.S.); jemina01@skku.edu (S.S.); 2 School of Mechanical Engineering, Sungkyunkwan University, Suwon 16419, Korea; whwlsdlf94@skku.edu (J.C.); choish3877@skku.edu (S.C.); kitakoo@skku.edu (K.P.); cmkim3@skku.edu (C.K.); 3 Department of Plasma Engineering, Korea Institute of Machinery & Materials (KIMM), Daejeon 34103, Korea; 4 Department of Materials Science and Engineering, Chungnam National University, Daejeon 34143, Korea; njeon@cnu.ac.kr

**Keywords:** chromism, strain sensor, pressure sensor, biosensor, energy storage, gasochromic, ion sensor, motion sensor

## Abstract

The bifunctionality of chromism-integrated sensors and devices has been highlighted because of their reversibility, fast response, and visual indication. For example, one of the representative chromism electrochromic materials exhibits optical modulation under ion insertion/extraction by applying a potential. This operation mechanism can be integrated with various sensors (pressure, strain, biomolecules, gas, etc.) and devices (energy conversion/storage systems) as visual indicators for user-friendly operation. In this review, recent advances in the field of chromism-integrated systems for visual indicators are categorized for various chromism-integrated sensors and devices. This review can provide insights for researchers working on chromism, sensors, or devices. The integrated chromic devices are evaluated in terms of coloration-bleach operation, cycling stability, and coloration efficiency. In addition, the existing challenges and prospects for chromism-integrated sensors and devices are summarized for further research.

## 1. Introduction

Chromic devices, which reversibly change their optical properties depending on the applied stimulus [1], have been studied for use in smart windows and glasses [2], information encryption [3], energy storage/conversion [4,5], and wearable sensors and electronics [6]. The special functionality of chromic materials can provide user-friendly visual detection and reversible fast response by coloration under the desired stimulus when integrated with another application [7].

For example, electrochromic devices (ECDs), which are representative chromic devices, are designed using multilayer structures consisting of a transparent conductor, an electrochromic (EC) layer, an ion conductor, an ion storage layer, and another transparent conductor. The EC material reversibly changes its optical properties via ion insertion/extraction under an applied potential [8]. Transition metal oxides (WO_3_, MoO_3_, V_2_O_5_, TiO_2_, Ta_2_O_5_, CeO_2_, etc.) [9,10], Prussian blue [11,12], and conducting polymers (polyaniline (PANI), poly(3,4-ethylenedioxythiophene (PEDOT), polypyrrole, etc.) [13,14] are selected because of their dramatic optical modulation under ion insertion/extraction. For the ion storage layer, the charge shuttled from the EC material must be balanced by the ion conductor [15]. Between the EC material and ion storage layer, an ion-conducting transparent electrolyte (liquid or solid) is positioned as the ion path [16]. In the transparent conductors at the end sides, fluorine- or indium-doped tin oxide (FTO and ITO)-coated glasses are used as electrodes. For example, the EC mechanism of WO_3_ (the most common EC material) can be described by the following reaction (M^+^: ion in the electrolyte):

WO_3_ (colorless) + x(M^+^ + e^−^) ↔ M_x_W^VI^_(1−x)_W^V^_x_O_3_ (blue) (1)

WO_3_ naturally exists in a bleached state, but it changes to blue (coloration) when ion insertion partially generates W^VI^ during the reduction process. This process is reversible under various applied potentials (bleached state ↔ colored state). 

**Scheme 1:**
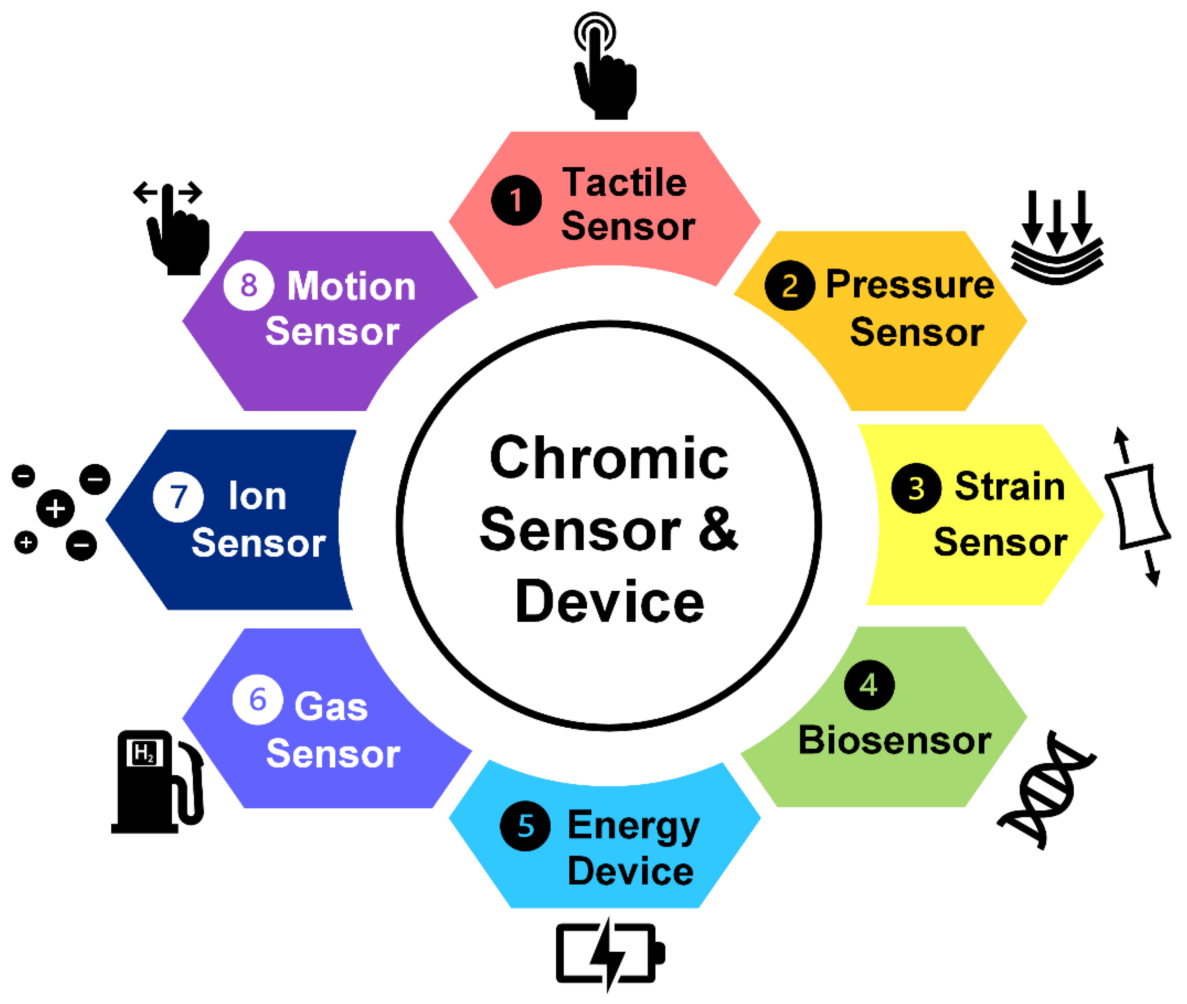
Schematic illustration of the chromic-system (visual indicator)-integrated sensors and devices.

The nanostructured EC materials can shorten ion diffusion length in electrolytes and facilitate accessibility in EC materials with large specific surface area to enhance the performance of ECDs. Additionally, the crystallinity of the EC materials provides increased intercalation sites for the ion in electrolytes. Owing to the fast ion insertion/extraction, long-term stability of ECDs with high-performance can be achieved by prohibiting trapped ions in the EC materials. Li et al. reviewed two-dimensional materials (2D) based on electrochromic applications showing superior electrochemical activity, fast charge transfer, and unique physical properties. Various 2D materials, such as metal oxide/dichalcogenides/nitrides, conductive polymer, and metal-organic frameworks are applicable for the ECDs owing to their advanced properties (flexible, transparent, and conducting 2D layers) [17]. In this regard, with the advantages of chromic devices (reversibility, fast response, and optical modulation), integration with other applications can be a synergistic combination, such as a visual display for various types of sensors (strain, pressure, biomolecules, gas, etc.) and energy storage/conversion-level indicators. Here, we introduce chromic-device-integrated sensors and devices as visual indicators, as shown in Scheme 1.

## 2. Tactile and Pressure Sensor Integrated Electrochromic Visual Detection 

Pressure sensing is a crucial function in various interactive devices, such as wearable devices [18], artificial prosthetics [19], smart robots [20], and e-skin [21]. A visually displayable e-skin integrated with electrochromic materials has a low-power consumption system and information display.

**Figure 1:**
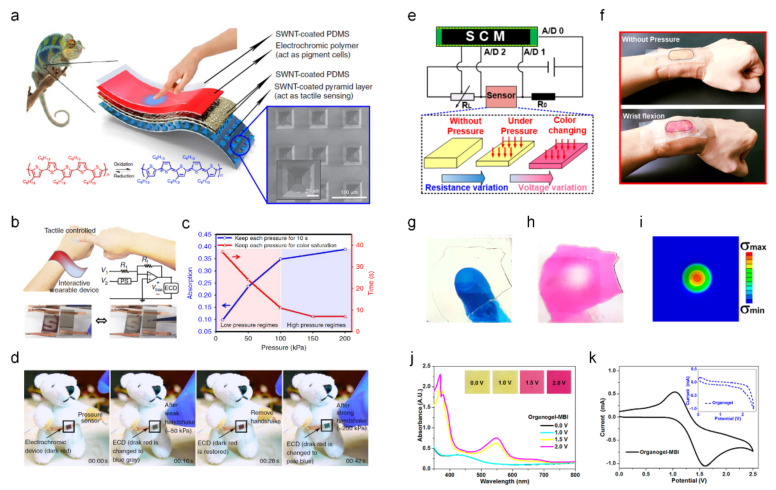
Wearable electrochromic devices (ECD) based on color-changeable e-skin integrated with a tactile sensor ((**a**–**d**) adapted with permission from ref. [22], copyright 2015 Nature Publishing Group and (**e**–**k**) adapted with permission from ref. [23], copyright 2020 American Chemical Society). (**a**) Chameleon-inspired e-skin by electrochromic polymer in poly (3-hexylthiophene-2, 5-diyl) (P3HT) with single-wall carbon nanotubes-coated pyramid layer of the tactile sensor (inset SEM image). (**b**) Schematic layout of the interactive color-changeable e-skin with the circuit and photos of PSEC. (**c**) The adsorption and time versus the low-pressure and high-pressure regimes of the designed e-skin. (**d**) Interactive color-changing and tactile-sensing e-skin. Depending on the strength of the handshake (tactile sensing), the color of the ECD changes (visual detection). (**e**) The circuit diagram with the working mechanism of the EC-tactile sensor for direct visualization of the stresses. (**f**) Interactive color change of ionic polyacrylamide (PAAm) organogel depending on the wrist flexion. (**g**) Pressing the PAAm organogel by finger and (**h**) direct stress distribution caused by the finger press. (**i**) Finite element analysis simulation of the stress distribution. (**j**) UV-vis absorption spectra with an optical image (inset) of the ionic PAAm organogel under varied potentials. (**k**) Cyclic voltammetric diagram of the PAAm organogel with or without (inset) 1-methyl-4,4’-bipyridinium iodide.

Chou et al. developed an ECD-integrated tactile sensor [22]. The detected pressure was visualized through the real-time coloration of the electrochromic materials. They are inspired by some animals, such as cephalopods or chameleons, which can change their skin color to mimic the properties of visually displayable e-skin fabrication. They demonstrated an all-solution processed stretchable e-skin with coloration functions by varying the applied pressure. Chameleon-inspired ECDs with pressure-sensitive tactile sensor components are illustrated in Figure 1a. As an electrochromic (EC) organic material, Poly (3-hexylthiophene-2, 5-diyl) (P3HT) is utilized by changing its color in a redox reaction. For efficient tactile sensing, spray-coated single-wall carbon nanotubes were uniformly deposited on top of the pyramidal-microstructured polydimethylsiloxane (PDMS) surface. Figure 1b shows a schematic of the interactive color-changeable e-skin with the circuit and photo of the ECDs. With various applied pressures (tactile sensing), the integrated ECD components visually display the pressure level. The absorption and time (s) depending on the pressure regime are plotted in Figure 1c. In the low-pressure regime (0–100 kPa), the designed ECD-integrated tactile sensor takes a long time for color saturation with a larger absorption change, whereas in the high-pressure regime (100–200 kPa), light absorption changes and quick color saturation times are observed. Consequently, the designed ECD-integrated tactile sensor sensitively detects low pressure with dynamic absorption changes. For example, an applied pressure of 200 kPa requires 7 s to reach color saturation, whereas a pressure of 10 kPa requires 37 s for color saturation. The ECD-integrated tactile sensor shows an interactive reversible operation between the applied pressure and coloration, as shown in Figure 1d. It shows the tunability of the skin color interactive with the applied pressure for informative functions in e-skin applications. Upon applying a weak handshake (~50 kPa), the interactive ECDs exhibited a color change from dark red to blue-gray. Upon removing the applied pressure, the ECDs turned dark red, indicating their reversible properties. When a strong handshake (~200 kPa) was applied to the tactile sensor, the color of the ECDs changed to pale blue. This demonstrates the feasibility of the information expression of the detected pressure from EC coloration.

Liu et al. reported an ionic polyacrylamide (PAAm) organogel for simultaneous tactile sensing and interactive color change [23]. The PAAm organogel exhibited excellent piezoresistive behavior owing to the incorporation of LiCl. In addition, replacing the water in the PAAm hydrogel with dimethyl sulfoxide (DMSO) enabled electrochromism in PAAm. The designed ECD-integrated tactile sensor showed pressure-sensing interactive color-changing, exhibiting an extreme stretchability of 1600% of the elongation, 7.2 kPa of compressive modulus, an excellent transmittance up to 90%, and 0.5 s of coloration time. The circuit diagram with the working mechanism of the tactile sensor with the ECDs for direct visualization of the pressure is illustrated in Figure 1e. The resistance of the PAAm organogel changed depending on the applied pressure, resulting in voltage and color changes. As shown in Figure 1f, the designed ECD-integrated tactile sensor could fully conform to human skin, owing to its superior properties (stretchability, transparency, and super-softness). In addition, it demonstrated human body motion traces, such as wrist flexion and walking, for visualization. The images in Figure 1g–i show a visualization of the stress distribution. Upon pressing the PAAm organogel with one finger (Figure 1g), the color changed to express the stress distribution (Figure 1h). The simulated results of the stress distribution by finite element analysis were conducted, as shown in Figure 1i. The UV-vis absorption spectra and optical images of the PAAm organogel coloration under different voltages are shown in Figure 1j. Depending on the voltage, the PAAm organogel gradually changed from light yellow (0 V) to red (2.0 V). To elucidate the EC mechanism of the PAAm organogel, cyclic voltammetry (CV) of the PAAm organogel with or without (inset) 1-Methyl-4,4′-bipyridinium iodide (MBI) was investigated, as shown in Figure 1k. Two peaks (1.2 V and 1.6 V) observed in the case of the PAAm organogel–MBI correspond to the redox reactions for the color change.

Visual display of the pressure information integrated with the ECDs is promising for applications in visually interactive wearable devices, smart robots, artificial prosthetics, etc.

**Figure 2:**
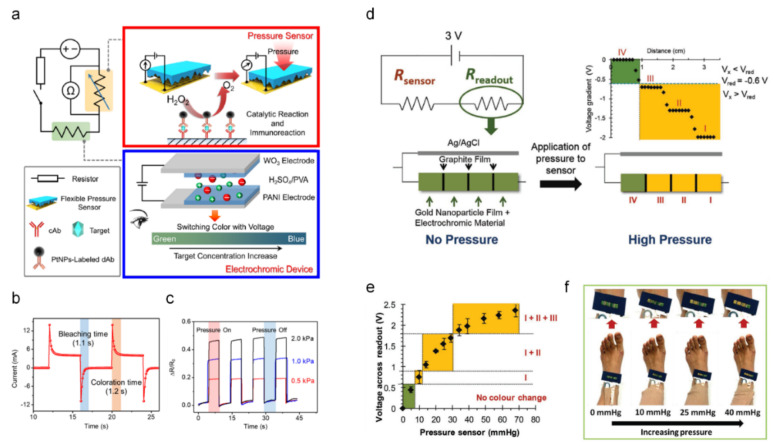
Pressure sensor integrated with EC visual detection ((**a**–**c**) adapted with permission from ref. [24], copyright 2021 American Chemical Society and (**d**–**f**) adapted with permission from ref. [25] copyright 2016 Wiley-VCH Verlag GmbH & Co.). (**a**) Schematic circuit diagram of the pressure-based immunoassay platform; flexible pressure sensor by catalytic reaction and immunoreaction (red box) and voltage-regulated ECD as a visualized readout (blue box). (**b**) Color switching time of the ECD. (**c**) Pressure response of the designed skin-inspired pressure sensor. (**d**) Operating mechanism of the paper-based ECD is incorporated with a pressure sensor (Rsensor). The paper-based readout system consists of resistive graphite separators and gold nanoparticle segments (readout) with Prussian blue/polyaniline as electrochromic materials. (**e**) Visual readout of pressure vs. voltage by a gradual color change of the gold nanoparticle segments. (**f**) A pressure readout system was applied to the bandage at the ankle.

Yu et al. demonstrated a portable pressure-based immunoassay integrated with the ECDs for visual detection [24]. The operating mechanism of the designed system is shown in Figure 2a. For the pressure sensor (red box), the reaction of platinum nanoparticles with hydrogen peroxide (H_2_O_2_) increases the pressure owing to a large volume change from liquid (H_2_O_2_) to gas (O_2_). In addition, a human-skin-inspired flexible sensor sensitively detects the pressure signal. In the ECD part (blue box), the ECDs provide a visual readout of pressure variance. As the applied pressure increases, the number of conductive paths increases, and thus, the resistance of the system decreases. Based on the electrical signal from the pressure sensor, the ECDs changed their color from green to blue for a visual readout of the pressure level. In Figure 2b, the color-switching time of the designed ECDs was investigated by chronoamperometry, exhibiting 1.1 s of bleaching and 1.2 s of coloration time. The real-time pressure response of the human-skin-inspired pressure sensor was characterized by investigating the resistance variance depending on the applied pressure, as shown in Figure 2c.

Liana et al. developed a simple and portable integrated platform for bandaging using piezoresistive pressure with the ECDs for a visual readout of the applied pressure [25]. A graphite pencil was used to tune the resistance of the system for the coloration of the ECDs, working as a visual readout of the applied pressure. The basic operating mechanism of the designed piezoresistive pressure-sensor-integrated ECD system is illustrated in Figure 2d. A paper-based readout system was designed using a gold nanoparticle-coated film with EC Prussian blue/polyaniline and graphite films (as resistive material separators). When a voltage is applied, the significant voltage drop at the resistive graphite separators induces different potentials for each gold nanoparticle segment. Therefore, gradual colorations of the paper-based ECDs provide an easy and quick interpretation of the applied pressure for the end user. The pressure applied to a piezoresistive sensor affects R_sensor_; thus, the potential at the readout varies, resulting in gradual coloration at the readout of the pressure level. In Figure 2e, depending on the applied pressure, each segment shows a gradual color change with an increased voltage readout. It was demonstrated as a bandage around the ankle, showing practical application for home-care services (Figure 2f). Upon increasing the pressure from 0 to 40 mmHg, signals were converted from the piezoresistive pressure sensor to the ECDs for gradual coloration, indicating the pressure level. This piezoresistive pressure sensor integrated with the ECD system suggests a new paradigm shift of EC wound care from outpatient services to personalized home care.

## 3. Electrochromic Integrated Strain Sensor for Visualization

A skin-attached stretchable strain sensor can detect various body motions by converting an applied force into an electrical signal [26,27]. Various studies have been conducted on stretchable strain sensors using piezoresistive materials [28,29]. To obtain information directly, visual detection of the body motion from strain sensors integrated with ECDs should be realized.

**Figure 3:**
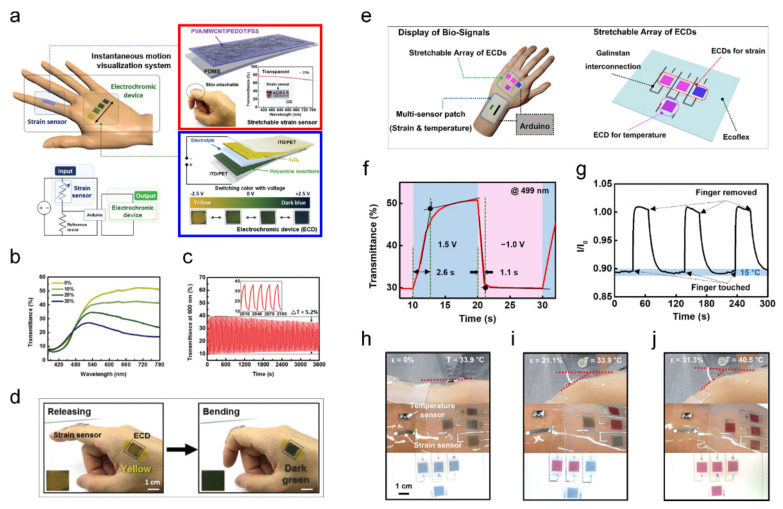
Electrochromic integrated strain sensor for visualization ((**a**–**d**) adapted with permission from ref. [30], copyright 2017 The Royal Society of Chemistry, and (**e**–**j**) adapted with permission from ref. [31], copyright 2022 Elsevier). (**a**) Schematic and circuit diagram of an interactive color-changeable platform with an ECD integrated strain sensor. Strain sensors are composed of the PVA/MWCNT/PEDOT:PSS on a PDMS substrate with a transmittance spectrum (red box). ECD consists of a polyaniline nanofiber/electrolyte/V_2_O_5_ with an ITO-coated PET film as an electrode, displaying a color change from yellow to dark upon application of a voltage (blue box). (**b**) Transmittance change under varied applied strain (0, 10, 20, and 30%). (**c**) Current-voltage curves of the strain sensor under various strain (10, 15, 20, and 30%). (**d**) Photograph of the ECD integrated strain sensor with finger motions. (**e**) Skin-attachable ECD array with strain and temperature sensor integration (**left**) and a stretchable array of ECDs (**right**). (**f**) Transmission change of the designed ECD under bias voltages at 499 nm. (**g**) Normalized current (I/I_o_) variation by finger touch. Visual information of wrist bend and skin temperature from the skin-attached array of ECD color patterns varying under the applied strain (ε) and temperature (T): (**h**) T = 33.9 °C; ε = 0%, (**i**) T = 33.9 °C; ε = 21.1%, (**j**) T = 40.5 °C; ε = 31.3%.

Park et al. developed a skin-integrated transparent and stretchable strain sensor with interactive color-changing ECDs, the schematic of which is illustrated in Figure 3a [30]. For the strain sensor, poly(vinyl alcohol)/multi-walled carbon nanotube/poly(3,4-ethylenedioxythiophene):poly(styrenesulfonate) was placed on a polydimethylsiloxane (PDMS) substrate, which was transparent and skin attachable for body motion detection (red box). ECDs were fabricated using polyaniline nanofibers and V_2_O_5_ on an indium-tin-oxide-coated polyethylene terephthalate film, and the color changed from yellow to dark blue under an applied voltage (blue box). The ECD-integrated strain sensor on human skin enables interactive real-time visual detection of body motion. In Figure 3b, the transmittance change is characterized depending on the applied strain (ε) from 0% to 30%. As the strain is increased, the transmittance gradually decreases, resulting in a color change from yellow to dark blue in the ECDs. Repetitive measurements of the relative resistance (ΔR/R_o_) for different applied strains from 10% to 30% showed reliable performance. A real e-skin demonstration of the ECD-integrated strain sensor for detecting finger bending is shown in Figure 3d. An interactive electrical signal with color-changing properties between the strain sensor and ECD is operated depending on finger motion. In addition, the color change is reversible, releasing (yellow) and bending (dark green). This ECD-integrated strain sensor shows gauge factor (ΔR/R_o_/ε) of 5.2 up to 50% strain, transparency of 77%, a fast response time of 20 ms, and high robustness and durability over 10,000 stretching/releasing cycles. In addition, various body motions, including biosignals, can be visually detected.

Kim et al. fabricated an interactive display system consisting of a stretchable array of ECDs and temperature and strain sensors for the visual detection of skin temperature and body motion [31]. A schematic of the designed platform is shown in Figure 3e. For the bio-signal display, the strain sensor was fabricated by embedding fragmentized graphene foam into a PDMS film to form a conductive network to detect the resistance change in body motion. In the construction of the temperature sensors, polyaniline (PANI) and multi-walled carbon nanotubes were embedded into poly (vinyl alcohol) (PVA), which shows a linear resistance change with temperature. A stretchable array of ECDs was composed of P3HT and tungsten trioxide (WO_3_) nanoparticles as electrochromic materials and Li-mixed 1-butyl-3-methylimidazolium bis(trifluoromethylsulfonyl)imide/poly(methyl methacrylate) ([BMIM][TFSI]/PMMA) as the electrolyte. Depending on the oxidation process, the P3HT conducting polymer changes its color from magenta to pale blue. WO_3_ is a representative inorganic electrochromic material with excellent color contrast and chemical stability. WO_3_ is utilized as an ion storage layer in ECDs because of its ability to store Li^+^. The designed ECDs exhibited a low power consumption of 65 μW/cm^2^ at −1.0 V, a high coloration efficiency of 448.1 cm^2^/C, and high chemical and mechanical stability. The real-time transmittance change in the designed ECDs under an applied voltage was investigated, as shown in Figure 3f. The ECDs take 2.6 s (1.5 V) for the color to change from magenta to pale blue, and 1.5 s (−1.0 V) for the color to change from pale blue to magenta. In addition, the normalized current (I/I_o_) of the designed temperature sensor was characterized by finger touch, exhibiting repetitive temperature sensing, as shown in Figure 3g. In Figure 3h–j, the simultaneous sensing of temperature and strain interaction with ECDs is demonstrated on the human wrist. In the case of no wrist bending (ε = 0%) and normal human temperature (33.9 °C), blue colors were observed on the three ECDs integrated with a strain sensor and the ECD integrated with a temperature sensor (Figure 3h). After wrist bending (ε = 21.1%), the three ECDs integrated with the strain sensor gradually changed their color from blue to magenta (Figure 3i). In the case of increased human skin temperature (40.5 °C) and wrist bending (ε = 31.3%), all the integrated ECDs showed color-changing from blue to magenta (Figure 3j). Table 1 summarizes various type of ECDs integrated sensors and devices to provide comprehensive understanding.

**Table 1:** Various ECD integrated sensors and devices.

Chromic Material	Color Display	λ (nm)	ΔT_b-c_ (%)	Applied Voiltage [V]	Response Time [s] Coloration Bleaching		Reference
P3HT/SWNT film	dark red ↔ pale blue	550	55	−1.0 ↔ +1.0	1.4	1.2	[22]
covalent organic framework	black ↔ transparent	574	33	−1.8 ↔ +2.0	1.8	2.6	[32]
W/WO_3_-V_2_O_5_	yellow ↔ dark blue	560	77.50	−1.5 ↔ +1	68.7	33.3	[33]
MnO_2_-encrusted V_2_O_5_ nanowires	blue ↔ green ↔ orange	550	31	−3.0 ↔ −2.5 ↔ +3.0	1.6	-	[34]
DHPV with (PVB)-carbonate	transparent ↔ deep blue	599.5	50.90	0 ↔ −1.0	5.6	5.3	[35]
polyimide + TAA with PI-1a	transparent ↔ black	798	96.80	0 ↔ +1.3	1.3	1.1	[36]
DFTPA-PI-MA film	yellow ↔ olive green	905	90	−0.25 ↔ +1.25	5.3	12.2	[37]
W_18_O_49_ nanowire thin film	transparent ↔ deep blue	632.8	50	−1.0 ↔ +1.0	2	2	[38]
Di-alkynyl substituted viologen	transparent ↔ blue	605	74.30	+1.6 ↔ −0.3	2.8	2.4	[39]
xanthommatin	red ↔ yellow	555	21.50	−1.5 ↔ +1.5	2.1	3.6	[40]
tetraphenylbenzidine/ Sb-doped SnO_2_	transparent ↔ black	605	57.82	0 ↔ +1.5	0.5	0.6	[41]
2D TiO_2_/Ti_3_C_2_T_x_ heterostructures	transparent ↔ opaque blue	550	41.09	0 ↔ −1.6	1.63	0.38	[1]
Zn–SVO	orange ↔ yellow ↔ green	632.8	21	+0.2 ↔ +1.2 ↔ +2.0	12.6	25.4	[42]
WO_3_, (PEDOT: PSS)	transparent ↔ blue	670	87	−0.5 ↔ +2.5	0.7	7.1	[43]
EV-Graphene nanoflakes (GNFs)-TFT	transparent ↔ blue	590	49	0 ↔ +1.8	0.4	0.9	[44]

## 4. Visual Biosensing System Based on the Electrochromic Readout

Biosensing platforms for addressing upcoming issues (airborne infection, real-time diagnosis, sensitivity, and simultaneous detection) have been intensively studied [45,46,47]. In this study, we introduced biosensor platforms integrated with colorimetric readouts that provide visual signals. The biosensing system is considered a highly advantageous platform because it does not require an additional detector or information display. In this regard, the reversible EC property, simple and fast response, and visual display are suitable for integration with a biosensor.

**Figure 4:**
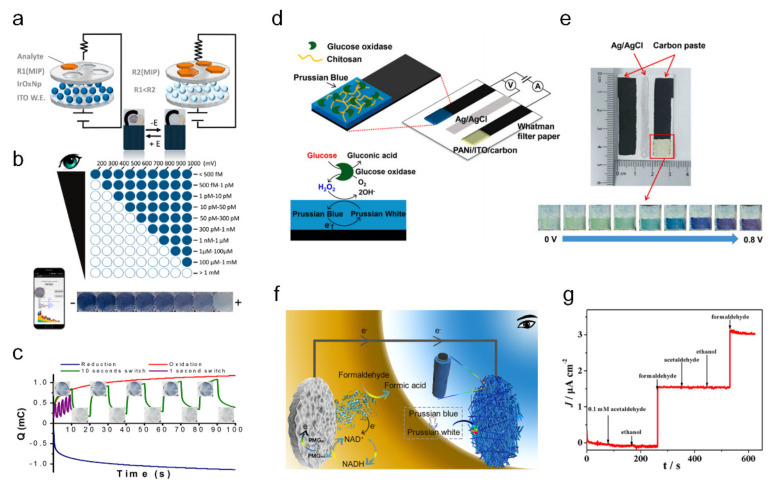
Visual biosensing system based on the EC readout ((**a**–**c**) adapted with permission from ref. [48], copyright 2018 American Chemical Society, (**d**,**e**) adapted with permission from ref. [49], copyright 2022 Elsevier, and (**f**,**g**) adapted with permission from ref. [50], copyright 2019 American Chemical Society). (**a**) Schematic of the IrO_x_ NPs electrochromism induced by the change in resistance in molecularly imprinted polymer due to chlorpyrifos analyte. (**b**) Visual detection of chlorpyrifos under varied oxidation potentials and chlorpyrifos concentrations. (**c**) Charge (mC) change of the IrO_x_ NPs during 100 s of oxidation (red), reduction (blue), and redox cycles with a switch of 1 s (purple) and 10 s (green). (**d**) The schematic and operating mechanism of the paper-based electrochromic glucose sensor. (**e**) Photographs of the working, reference, and counter electrodes (from left). EC behavior of the deposited PANI under different potentials (blue box). (**f**) Working principle of the enzymatic self-powered biosensor (ESPB) in formaldehyde detection. (**g**) Short-circuit current of the ESPB by successive addition of 0.1 mM acetaldehyde, 0.1 mM ethanol, and 0.1 mM formaldehyde, which indicates selectivity toward formaldehyde.

Capoferri et al. developed an EC-integrated biosensor and a smartphone-based detecting platform [48]. For biomimetic sensors, molecularly imprinted polymers (MIPs), which have superior robustness, versatility, and chemical and thermal stabilities, are utilized for analytical applications. For the EC material, iridium oxide (IrO_x_) is exploited for its reversible and persistent color change by redox reaction from blue–black to transparent under various potentials. Capoferri et al. integrated this novel sensor with a smartphone to develop lab-on-smartphone platforms, such that real-time quantitative information on biochemical and EC detection could be provided to the end user. A schematic of the MIP/IrO_x_ nanoparticle (NP)-ITO screen-printed electrode structure and the color change of the IrO_x_ NPs from blue-black to transparent is illustrated in Figure 4a. Visual detection after 10 s at different oxidation states derived by varying potentials and concentrations was characterized, and smartphone-based detection of chlorpyrifos was achieved by image processing of the color intensity (Figure 4b). The charge (mC) of the oxidation (red) and reduction (blue) states of the IrO_x_ NPs were measured, as shown in Figure 4c. Oxidation (+0.5 V)/reduction (−0.4 V) cycles of the IrO_x_ NPs were characterized by 10 s (green) and 1 s switching (purple), showing a repetitive coloration response. In this study, they developed an EC-integrated portable and user-friendly biosensor platform for in situ visual detection.

Yeon et al. designed a paper-based EC glucose sensor using redox-reaction-derived colorimetric visual detection [49]. Figure 4d shows the schematic of the paper-based EC glucose sensor platform, where the sensing mechanism is illustrated. Prussian blue and glucose oxidase (GO_x_) were used for the hydrogen peroxide (H_2_O_2_) and glucose detection. Colorimetric visualization is displayed at the counter electrode (PANI on the ITO-modified carbon electrodes). Under an applied potential at the working electrode, the current generated from the redox reaction alters the potential on the counter electrode, which is the EC readout of the color change under varied potentials. The optical properties change depending upon the applied potential on the optical readout section from light yellow (0 V) to purple (0.8 V), enabling visual detection of the bio-signal information (Figure 4e). The designed sensor exhibited a short response time (30 s) and glucose detection limit of 126 μM. The paper-based colorimetric sensor concept provides several advantages, such as cost-effectiveness, large-scale fabrication, and visual detection, in realizing point-of-care devices.

Sun et al. developed an enzymatic self-powered biosensor (ESPB) for formaldehyde detection [50]. A schematic of the designed system and its operating mechanism is illustrated in Figure 4f. The ESPB is composed of a formaldehyde dehydrogenase/poly methylene green/buckypaper bioanode, which serves as the sensor, and a Prussian blue/Au nanoparticle/carbon fiber paper cathode, which serves as the ECD. The successful detection of formaldehyde by the ESPB was achieved by a redox reaction in the bioanode and cathode. Prussian blue was changed to Prussian white when the electron was transported through an external circuit, leading to reduction. Formaldehyde worked as the fuel in the ESPB, and the detection limit of the sensor was 0.006 mM, showing linear detection properties from 0.01 to 0.35 mM. Additionally, the ESPB preserved 92% (after 21 days) and 80% (after 31 days) of the circuit currents compared with the original performance, suggesting long-term storage stability. The selectivity of the ESPB was investigated by the addition of acetaldehyde, ethanol (no current change), and formaldehyde (sudden current increase), as shown in Figure 4g. This colorimetric ESPB system can replace disposable test paper as a sensitive, simple, cost-effective, and high-selectivity system.

## 5. Electrochromic Energy Storage Devices 

ECDs reversibly change their optical properties under applied potentials with the redox process between the EC material and electrolyte [51]. During the redox process, ion insertion/extraction occurs in the EC materials, functioning as energy storage and conversion [52]. In recent years, these bifunctional devices (electrochromism and energy storage/conversion) have attracted attention for integrated applications in smart glasses and windows [53,54], wearable electronics [55], low-voltage displays [56], and self-powered electrochemical devices [5,57]. One obstacle for electrochromic battery is balanced between energy storage performance and electrochromic performance, in terms of electrode bulk. To achieve high power density, it is important to increase the volume of electrodes for more ion intercalation. However, in electrochromic devices, thick and bulky electrodes hinder fast response and lower transparency in the bleached state. Further research is necessary to achieve a solution for both functions.

**Figure 5:**
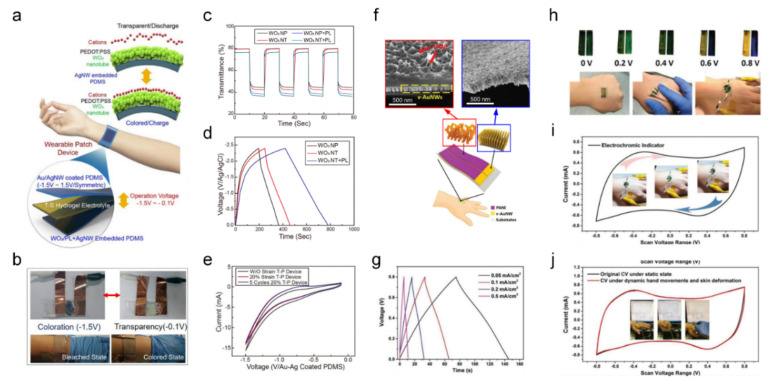
EC energy storage devices with the capability of visual charge level inspection ((**a**–**e**) adapted with permission ref. [6], copyright 2019 American Chemical Society, (**f**–**j**) adapted with permission ref. [58], copyright 2018 Wiley-VCH Verlag GmbH & Co.). (**a**) Operation schematic of an all-transparent stretchable EC supercapacitor (all-TSES). (**b**) Coloration (discharged state) and bleaching (charged state) of the all-TSES under normal and stretched states. (**c**) Transmittance change of various WO_3_ nanostructures consisting of the all-TSES. (PL: PEDOT: PSS Layer) (**d**) Galvanostatic charge–discharge (GCD) graphs of the all-TSES with three variations of the nanostructure combination (**e**) CV result of 20% stretched all-TSES devices. (**f**) Structural diagram of a wearable EC supercapacitor and its vertical gold nanowire (v-AuNWs) structure evaluated by SEM. (**g**) GCD curves of a v-AuNW/PANI supercapacitor under varied areal current densities. (**h**) EC properties of v-AuNW/PANI-based supercapacitor with different charge levels and under conditions required for flexibility. (**i**) CV curve with the photograph of EC change. (**j**) CV curves of the v-AuNW/PANI supercapacitor comparing the dynamic condition with the static state.

Yun et al. demonstrated an all-transparent stretchable electrochromic supercapacitor (ECS) for wearable patch devices using Au/Ag core-shell nanowire-embedded PDMS, bistacked WO_3_ nanotube/PEDOT: PSS, and PAAm-based hydrogel electrolytes [6]. A schematic of the operating mechanism of the designed wearable ECS is shown in Figure 5a. The electrospun WO_3_ nanotube was coated with a thin PEDOT: PSS layer and drop-coated onto the Ag nanowire-embedded PDMS substrate. The fabricated all-transparent stretchable ECS wearable patch worked with a coloration device and an electrochemical energy storage device. The electrochromic active material (WO_3_) composites and Au/Ag core-shell nanowire networks were sandwiched between the hydrogel electrolytes. The hydrogel electrolyte provided a conducting path for Li^+^ ion diffusion between the two separate electrodes. The reversible coloration (−1.5 V) and bleached (−0.1 V) states of the fabricated ECS were tested, as shown in Figure 5b. In Figure 5c, the synthesized WO_3_ nanoparticles and nanotubes coated on the Ag nanowire-embedded PDMS and PEDOT:PSS layer (PL) were tested for in situ transmittance variation between the bleached (T_b_) and coloration (T_c_) states at 635 nm. The measured coloration contrasts (T_b_-T_c_) were 35.3% for the WO_3_ nanoparticles and 37.7% for the WO_3_ nanotubes. As shown in Figure 5d, charge–discharge tests for all-transparent stretchable ECSs were conducted at a current density of 4 A g^−1^. A less steep discharge slope indicates a higher capacity of the ECSs owing to the addition of the WO_3_ nanotubes and PL. The CV test of the fabricated ECSs under a strain of 20% confirmed the retention of 98.6% for the capacitance (Figure 5e). An all-transparent stretchable ECS is a suitable candidate for wearable energy storage applications.

An et al. developed a second-skin-like ECS using self-assembled vertical gold nanowires (v-AuNWs) and electrodeposited PANI [58]. PANI works as an electrochromic material for energy storage-level indicators during the charge–discharge process. The v-AuNWs provided mechanical flexibility and deformability with enhanced conductivity in the ECS. The schematic and structural information of v-AuNW/PANI (red) and v-AuNWs (blue) are shown in Figure 5f. A second-skin-like conformable and multifunctional ECS consists of v-AuNWs (flexible and conducting material) and PANI (EC material). Galvanostatic charge–discharge tests were conducted under different areal current densities of the designed v-AuNW/PANI supercapacitors, as shown in Figure 5g. The ECS was successfully attached to a human hand with skin-conformability and retained its integrity under skin deformation (Figure 5h–j). In Figure 5h, PANI shows clear electrochromic properties under the charging–discharging process (0–0.8 V) on the human hand. A whole-range voltage sweep (−0.8 to +0.8 V) results in different oxidation states of PANI, which is required to investigate the electrochromic properties. Under the non-charged states, the ECS is green and varies under different potentials (blue at +0.8 V and yellow at −0.8 V), showing a reversible coloration process (Figure 5i). The fabricated second-skin-like ECS was investigated using CV curves under hand clenching or arbitrary skin deformations to confirm its skin-conformability and mechanical robustness, as shown in Figure 5j. The measured CV, before and after deformation was negligibly preserved and retained 99.5% of the capacitance. This second-skin-like ECS is an ideal candidate for future wearable on-skin electronics, owing to its bifunctionality in energy storage and coloration, superior skin conformability, and mechanical flexibility under skin deformations.

Furthermore, Kim et al. proposed single-layer ECSs (SL-ECSs) based on energy storage of EC ion gels and tailored diffusion dynamics for high-performance in ultra-compact functional electronics [59]. The diffusion coefficient and concentration gradient adjust the ability of energy storage. It shows high area capacitance (C_areal_) (≈ 43.0 mF cm^−2^), ΔT (96.8%), and capacitance retention over 80% after 3000 min operations. The practical multifunctionality with the superior performance SL-ECSs has been demonstrated as a power source and applied force monitoring platform.

## 6. Gasochromic Sensors

Gasochromism is a phenomenon that involves a colorimetric change upon exposure to a specific gas. Gasochromic sensors are being actively studied for the detection and visualization of the presence of gases. Hydrogen gas is a representative analyte. Because hydrogen is regarded as a promising future energy carrier, detecting the leakage of explosive hydrogen gas is of extreme importance and a key focus of research studies [60,61]. In this regard, a transition metal oxide, such as tungsten trioxide, is mainly used as a gasochromic material. With the fast and reversible intercalation of protons, WO_3_ exhibits active gasochromic behavior and good reversibility.

**Figure 6:**
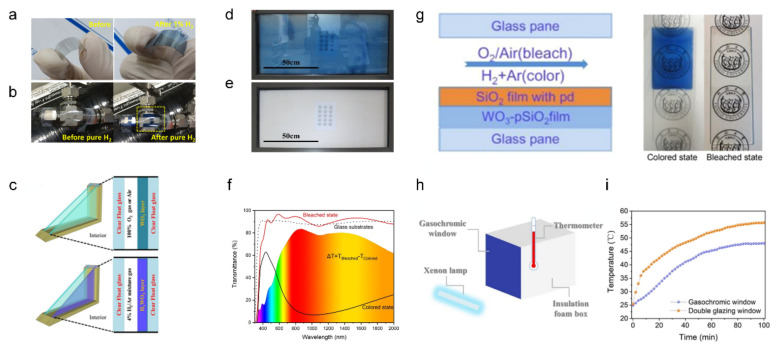
Gas sensor with chromic characterization ((**a**,**b**) adapted with permission from ref. [62], copyright 2017 Elsevier, (**c**) adapted with permission from ref. [63], copyright 2017 IOP Publishing, (**d**,**e**) adapted with permission from ref. [64], copyright 2022 Springer, (**f**–**i**) adapted with permission from ref. [65], copyright 2021 American Chemical Society). (**a**) Photographic images of Pd–WO_3_ films deposited on a flexible substrate for H_2_ detection: (**left**) pristine Pd-WO_3_, (**right**) color-changed Pd-WO_3_ under 1% H_2_ gas. (**b**) Photographic images of the installed Pd-WO_3_ films on a gas pipe carrying pure H_2_ before and after injecting H_2_. (**c**) Configuration of a gasochromic smart window. A large-scale gasochromic smart window (**d**) under colored and (**e**) bleached states. The area of the smart window was 1.3 × 0.8 m^2^. (**f**) Transmittance change of the WO_3_-SiO_2_ gasochromic films in the colored and bleached states. (**g**) Photographs of WO_3_-SiO_2_ in colored and bleached states. A schematic illustration of the assembled window is shown below. (**h**) Schematic of the insulated box for sunlight absorbance measurement. (**i**) Heating curve of the sunlight absorbance test.

Figure 6a shows photographs of the flexible WO_3_ hydrogen gas sensor and its electrochromic behavior under hydrogen exposure [62]. The high sensitivity of the sensor is a critical issue in H_2_ fuel applications because H_2_ gas exhibits explosive behavior at concentrations above 4% in air. To achieve high sensitivity, Lee et al. fabricated porous and amorphous tungsten oxide thin films induced by radio frequency (RF) sputtering and then doped catalytic palladium nanoparticles into a WO_3_ structure by e-beam evaporation. The fabricated cell displayed a high optical transmittance change of 50% after 10 min of exposure to 1% concentration of H_2_ gas. Furthermore, by wrapping the flexible ECD gas sensors around the gas pipeline, the practical application of visual gas leakage detection was demonstrated (Figure 6b).

Figure 6c shows a schematic of the gasochromic double-glazed unit (GC DGU) with a tungsten oxide nanoparticle film. Zhou et al. reported an economic template-assisted sol–gel method for the synthesis of WO_3_ nanospheres with a fast gasochromic response [63]. Owing to its porous microstructure and nanocrystalline nature, the fabricated WO_3_/Pd nanoparticle film has a large surface-active position of 166 m^2^g^−1^ and a shortened proton diffusion distance. After the GC DGU was exposed to 4% H_2_/Ar gas, the near-infrared (NIR) transmittance was remarkably reduced, while showing high transmittance of 75% in the visible spectra (380–780 nm). With this property, the GC DGU is expected to be applied not only to gasochromic sensors, but also as energy-saving heat control windows.

Gao et al. suggested a sol–gel method for the medium-scale production of gasochromic windows, as shown in Figure 6d [64]. By adequate reaction conditions, material ratio, and assemblage, a gasochromic window of area 0.8 × 1.3 m^2^ was prepared. Figure 6d shows the gasochromic window colored under hydrogen exposure and its bleached state (Figure 6e). The transmittance at 1000 nm was 22% in the colored state and 76% in the bleached state.

Gao et al. also reported a unique fabrication method for hydrogen detecting gasochromic windows by printing WO_3_–SiO_2_ ink [65]. With appropriate pH-value control on prehydrolysis of SiO_2_, a uniform and porous SiO_2_ supporting structure with WO_3_ was acquired. The gasochromic film obtained by dip-coating exhibited a high transparency of 80% transmittance in the bleached state and ΔT_c-b_ of 50%, as shown in Figure 6f. Figure 6g shows the schematics of the assembled window and colorimetric change to blue under hydrogen gas exposure. With respect to the energy-saving potential, the device proposed by this research group demonstrated superior infrared sunlight absorbance compared to that of commercial double-glazed windows, as evident from measuring the temperature of an insulated box with a xenon lamp of intensity 500 W/m^2^ applied at the 30 × 30 cm^2^ window side (Figure 6h). After 100 min of exposure, the temperature in the box with the gasochromic window was 47.9 °C, whereas the double-glazed window showed a significantly higher temperature of 55.8 °C, as shown in Figure 6i.

To enhance the gasochromic sensing performance, researchers have reported the use of noble metals as catalysts [66,67,68]. Under hydrogen exposure, the doped Pt group catalyst dissociated hydrogen molecules into hydrogen atoms, which migrated into the adjacent metal oxide for the reaction. This phenomenon is called the spillover effect [69,70,71,72,73]. With this spillover effect, gasochromic response and sensitivity to hydrogen could be enhanced.

**Figure 7:**
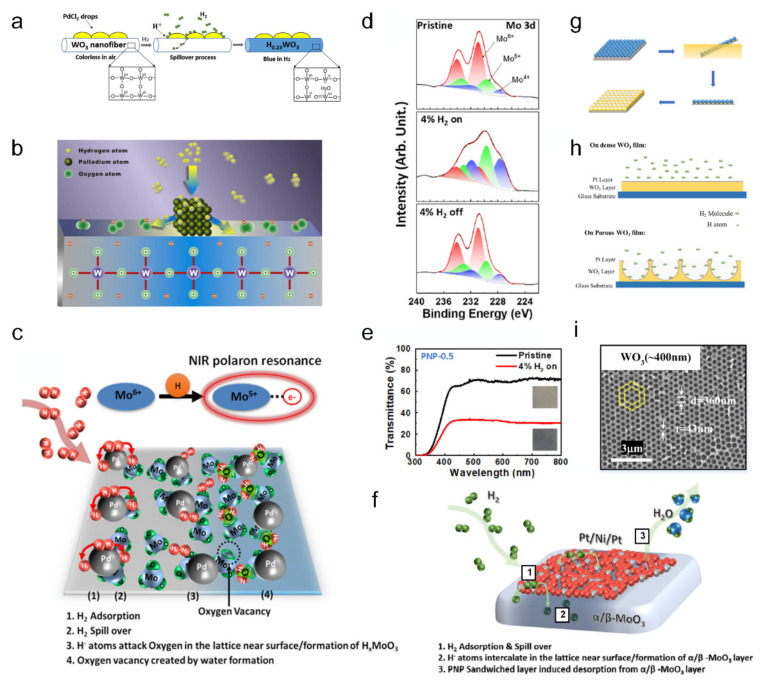
The gasochromic mechanisms of transition metal oxides with noble metal catalysts ((**a**) adapted with permission from ref. [74], copyright 2018 Elsevier, (**b**) adapted with permission from ref. [76], copyright 2014 Elsevier, (**c**) adapted with permission from ref. [77], copyright 2017 Elsevier, (**d**–**f**) adapted with permission from ref. [83], copyright 2021 Elsevier, and (**g**–**i**) adapted with permission from ref. [85], copyright 2022 Springer). The schematic of the mechanism for (**a**) a gasochromic PdCl_2_-WO_3_ nanofiber and (**b**) Pd-WO_3_ film with H_2_ molecule. (**c**) XPS spectra of the Pt/Ni/Pt-0.5/MoO_3_ film before, during, and after H_2_ exposure. (**d**) Visible spectra transmittance of the pristine WO_3_ (black line) and H_2_ exposure states. (redline) (**e**) The illustration of the H_2_ sensing mechanism for the Pt/Ni/Pt-0.5/MoO_3_ film. (**f**) Suggested H_2_ sensing mechanism for the Pt/Ni/Pt-MoO_3_ composite. (**g**) Schematic of the porous WO_3_ fabrication by the polystyrene template method. (**h**) Conceptual comparison between dense WO_3_ and porous WO_3_ films with H_2_. (**i**) SEM image of the fabricated porous WO_3_ film of 360 nm diameter.

Foroushani et al. demonstrated a gasochromic WO_3_ hydrogen sensor with an electrospun nanofiber web structure in a palladium chloride solution [74]. Figure 7a shows a schematic of the spillover process of the WO_3_ nanofiber hydrogen sensor. As the reduced palladium acts as a catalyst, the tungsten oxide nanofibrous webs show high hydrogen sensitivity of 2% concentration at room temperature, whereas the former electronic hydrogen sensor requires high temperature for detection [75].

Liu et al. also demonstrated an H_2_ gasochromic sensor using tungsten trioxide and the spillover effect induced by a palladium nanocube [76]. Figure 7b shows a conceptual diagram of the spillover effect of palladium nanoparticle on WO_3_ nanoplates. The Pd/WO_3_ composite accomplished a low detection limit of 0.05% H_2_/Ar. Furthermore, a fast response time of 21 s was achieved under 0.05% H_2_/Ar gas, verifying that it showed a faster response time (10 s) under a higher concentration (1%) of H_2_ gas. 

Kalanur et al. demonstrated a MoO_3_ nanoplate hydrogen sensor with doped palladium nanoparticles that showed stable irreversibility for a few weeks [77]. Figure 7c shows the proposed mechanism for the MoO_3_ gas sensor. They suggested that the irreversible chromic change of the sensor came from trapped H_2_O molecules, which were formed during the reaction between the dissociated hydrogen by the palladium and MoO_3_ lattice [78].

MoO_3_ + xH^•^ → H_x_MoO_3_ (2)

The principle of gasochromic color change induced by dissociated hydrogen atoms is based on ‘small-polaron resonance’ [79,80,81,82], leading to high sensitivity (functions at 0.1% H_2_ concentration) and long-term irreversibility. The proposed device is expected to be used as a printable sensor for outdoor usage with an adequate substrate, such as a polymer.

Han et al. fabricated a hydrogen sensor based on mixed-phase molybdenum oxide using a Pt-Ni-Pt (PNP) catalyst [83]. Unlike the aforementioned irreversible MoO_3_ hydrogen sensor, the mixed-phase PNP/MoO_3_ sensor exhibited a reversible behavior. Figure 7d shows the X-ray photoelectron spectroscopy (XPS) spectra of the PNP/MoO_3_ film in three steps: before H_2_ exposure (pristine state), during H_2_ exposure, and after ambient exposure. Owing to oxygen vacancies at 4% hydrogen exposure, the lower oxidation state of Mo becomes dominant, causing increased peaks of Mo^5+^ and Mo^4+^. The pristine and after ambient exposures were compared using the XPS results, and the reversibility of PNP/MoO_3_ was verified. A visible color change due to the hydrogen gasochromic reaction of the PNP/MoO_3_ film can be seen, as shown in Figure 7e. After the 4% H_2_ gas exposure, the optical transmittance dropped to 30%, whereas the transmittance of the initial pristine state was 70%. Figure 7f shows the interaction mechanism of PNP/MoO_3_ with H_2_ gas based on the spillover effect. The authors suggested that the combination of orthorhombic α-MoO_3_ and monoclinic β-MoO_3_ succeeded in acquiring the advantages of both phases, thereby exhibiting good gasochromic properties and thermodynamic stability [84]. Furthermore, by connecting the Ar/Cr electrode to the PNP/MoO_3_ films, the PNP/MoO_3_ film could detect hydrogen gas both optically and electrically.

Nie et al. reported an ordered porous WO_3_ film with sputtered Pt, which showed a faster response than a dense WO_3_/Pt film [85]. The porous WO_3_ structure was fabricated by dip-coating a self-assembled polystyrene template onto a WO_3_ solution, as shown in Figure 7g. The pore size of WO_3_ was optimized for gasochromic performance by controlling the diameter of the PS spheres. Owing to the spillover effect and highly active surface induced by the porous structure (Figure 7h), a 325 nm pore-sized WO_3_/Pt film (Figure 7i) could acquire 66.3 s of response time under 4% H_2_ exposure. Table 2 summarized hydrogen gas sensors with various fabrication method for chromic materials and ΔT_b-c_ at specific λ and detect environment.

**Table 2:** Various gasochromic system for hydrogen gas detection.

Chromic Material		Fabrication Method	λ (nm)	ΔT_b-c_ (%)	Gas	Reference
WO_3_	WO_3_-SiO_2_	Prehydrolyzing SiO_2_ + TEOS	1000	84	10% H_2_/Ar	[65]
	WO_3_ film	Prehydrolyzing SiO_2_ + dip coating (block copolymer)	1000	30	10% H_2_/Ar	[86]
	Pd/WO_3_/Graphene	Prehydrolyzing SiO_2_ + PdCl_2_ + spin coating	1000	72	4% H_2_/Ar	[87]
	WO_3_ film	RF magnetron sputtering + E beam evaporation	900	50	1% H_2_/Air	[62]
	WO_3_ film	Templated assisted peroxopolytungstic acid sol gel method	1000	65	4% H_2_/Ar	[88]
	WO_3_ film	Spin coating on PET + UV irradiation	1000	35	10% H_2_/Ar	[89]
	WO_3_/SiO_2_ + Pd film	Dip-coating in WO_3_ sol. And SiO_2_ + Pd film coated	1000	43	10% H_2_/Ar	[90]
	Ordered porous WO_3_	Colloidal template method + sputtering Pt	1500	28.60	4% H_2_/Ar	[85]
	PtNPs/PBNPs film	Sequential sipin coating of PBNPs & PtNPs on glass	700	81.50	4% H_2_/Ar	[91]
MoO_3_	Pt/Ni/Pt-MoO_3_	RF magnetron sputter + E beam evaporation	435	16	4% H_2_/Air	[83]
	MoO_3_ film	Electrodeposited Na_2_MoO_4_ solution on FTO	900	40	3% H_2_/N_2_	[84]
	Pd/MoO_3_	Hydrothermal synthesis + photochemical deposition of Pd	700	40	1% H_2_/N_2_	[81]
Ni based	NiOOH/Pd	NiOOH thin film by chemical bath deposition method	650	50.60	100% H_2_	[92]
VO_x_	Pt/Mo V_2_O_5_ films	Sol gel method spin coating with ion sputtering pt	750	20.30	1% H_2_/N_2_	[93]
	Pt/V_2_O_5_ films	Sol-gel spin coating	740	40	3.6 × 10^−7^ Pa H_2_	[94]
	Pt/V_2_O_5_ films	Sol gel method (Spin coating)	740	39	5 × 10^−7^ Torr H_2_	[95]
	Pt/VO_2_	Sol gel method (Spin coating)	400	50	2.7 × 10^−9^ Torr H_2_	[96]
Nb_2_O_5_	Pd/Mg Nb_2_O_5_ films	Magnetron sputtering + PECVD	940	45.30	4% H_2_/Ar	[97]
Polymer	PXDOTS	Anodic electrochemical polymerization	550	50.70	100% H_2_	[98]

## 7. Ion Sensors

The selective determination of heavy metal ions is a challenging problem [99], owing to their contribution to accumulating strong toxicity in the environment and human body [100]. An ionochromic device that shows reversible color change in the presence of specific ions is considered an intuitive solution for practical ion detection in various fields [101]. Since there are many ion-mediated chemical reactions, the selectivity of an ion sensor that only reacts with a specific ion is an important indicator. Besides selectivity, high sensitivity is another major performance of ion sensor.

**Figure 8:**
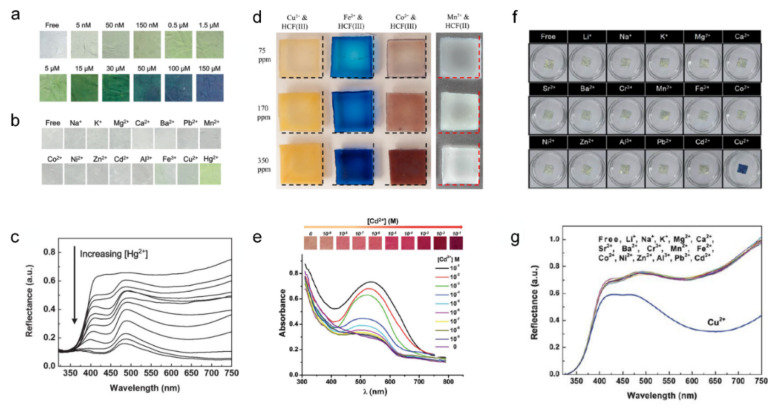
Ion responsive chromic device ((**a**–**c**) adapted with permission from ref. [102], copyright 2014 The Royal Society of Chemistry, (**d**) adapted with permission from ref. [103], copyright 2018 Elsevier, (**e**) adapted with permission from ref. [105], copyright 2014 The Royal Society of Chemistry, and (**f**,**g**) adapted with permission from ref. [106], copyright 2011 The Royal Society of Chemistry). (**a**) Gradual colorimetric change of the PANI-LB under Hg^2+^ exposure in an aqueous solution. (**b**) Selectivity test conducted by exposing the PANI-LB to 5 μM of various metal ion solutions. (**c**) Reflectance in visible spectra with increasing concentration of Hg^2+^. (**d**) Photographs of color-changed hydrogels exposed to various transition metal ions. Swelling and deswelling of the gels can be detected in comparison with the dash lines corresponding to 3 × 3 cm^2^. (**e**) Images of color gradient and UV-vis absorbance spectra of SiO_2_ and DPC-doped fibrous films under different Cd^2+^ ion concentrations. (**f**) Photographs and (**g**) reflectance spectra displaying the selectivity of the fabricated membranes in various metal ions with the concentration of 1 ppm.

As shown in Figure 8a–c, Si et al. reported a chromic sensor strip that is selective for mercury, a well-known toxic heavy metal [102]. This ionochromic sensor acquired its selectivity through the specific interaction of leucoemeraldine-based polyaniline (PANI-LB) with Hg^2+^ ions. With a nanofibrous structure fabricated by electrospinning, this mercury sensor exhibited high sensitivity with a low detection limit of 5 nM. Figure 8a shows the gradual color change from white to blue with increasing mercury ion concentrations up to 150 μM. The selectivity for mercury was evaluated by exposing the PANI-LBNF sensor to 5 μM of various metal ions for 20 min, as shown in Figure 8b. The measured reflectance in the visible spectra indicated a distinct colorimetric change (Figure 8c).

Price et al. evaluated the colorimetric sensing mechanisms of hydrogel compositions with polyacrylic acid and acrylamido-methyl propane sulfonic acid, which showed a chromic reaction with 11 different hexacyanoferrate ions [103]. Figure 8d shows various colorimetric changes in the hydrogel composition under different transition metal ions from the transparent initial state (copper: golden yellow, iron: Prussian blue, cobalt: reddish brown, manganese: cloudy white). Owing to the preconcentration of the analyte ions by the high charge density of the hydrogel headgroup, this ionochromic sensor achieved a low detection limit of 100 ppb. In addition, the fabricated hydrogel showed chemoresistive properties that could be adjusted by the gel dimensions. With this unique chromic property induced by the redox reaction of hexacyanoferrate [104], this ionochromic sensor is expected to be a versatile identification method for various metals.

Yao et al. also reported another colorimetric ion sensor for selective cadmium detection with an electrospun nanofiber structure by electrospinning polymethylmethacrylate (PMMA)/diphenyl carbazide (DPC) with doped SiO_2_ nanoparticle [105]. As shown in Figure 8e, a distinct colorimetric change from yellow to red was confirmed as the Cd^2+^ concentration increased. This was based on the formation of the Cd–DPC complex (shown in red). With the electrospun nanostructure, the PMMA/DPC cadmium sensor achieved a good sensitivity of 10^−8^ M limit of detection via a high specific surface area. Furthermore, demonstrating colorimetric changes with other metal ions showed distinct selectivity toward cadmium ions, which was essential as a cadmium sensor.

As shown in Figure 8f,g, Ding et al. demonstrated a flexible and ultrasensitive copper ion sensor based on a polyaniline/polyamide-6 (PANI/PA-6) nanofiber/net (NFN) structure [106]. Because the unique electrospun nanofiber/net structure had a high specific surface area, the PANI/PA-6 copper sensor exhibited ultra-sensitivity, which could be observed by the naked eye, even at a 1 ppb of Cu^2+^ concentration. With the selective redox reaction of the PANI-LB (PANI leucoemeraldine base) upon the Cu^2+^ ions, the fabricated PANI/PA-6 copper sensor turned blue only when copper ions were present (Figure 8f), owing to the distinct reflectance decrease at 435 nm and 650 nm (Figure 8g).

## 8. Motion Sensors

User-interactive displays, such as visualization of touch, human temperature, or humidity, are also one of the attractive fields where the chromic principle can be applied owing to their high potential for use in upcoming human-machine interface technology, such as patchable electronics (e-skin). Chromic visualization, however, has significant benefits in terms of power source, because it does not require an information-converting microprocessor or a high-power source for an electroluminescent component, such as an LED [107,108,109]. For colorimetric visualization, a structure called ‘photonic crystal’, which is a nanostructure with a periodic refractive-index change, has been used in the studies described below. With photonic crystals, a phenomenon called ‘structural coloration’ [110] is known to induce selective reflectance spectra even without the actual color of the nanostructure itself. Integrating this structural phenomenon with humidity-sensitive material could suggest one possible solution to the motion sensing method. By properly controlling the structure of the photonic crystals, the researchers have achieved a distinct chromic visualization of humidity caused by human finger motion in the visible spectra.

**Figure 9:**
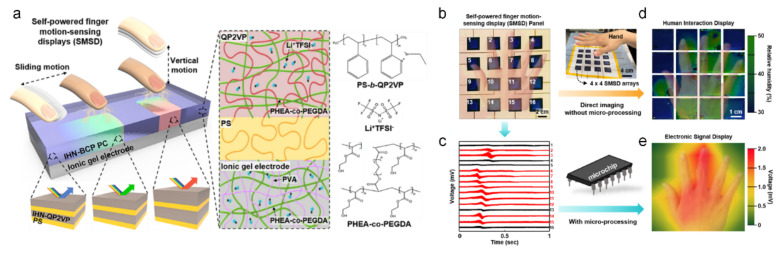
(**a**) Schematic illustration of the self-powered finger-motion-sensing display (SMSD) based on an IHN-BCP film on an ionic gel electrode, and touchless motion sensing showing the color change in the IHN-BCP layer. (**b**) The photograph of 4 × 4 arrays of the SMSDs on a flexible 20 × 20 cm^2^ substrate. (**c**) The voltage signals of the SMSD panel according to the hand motion. (**d**) Two-dimensional contour plot mapping of the voltage signal. (**e**) Images of the structural color of the IHN-BCP from touchless hand motion. (**a**–**e**) adapted with permission from ref. [111], copyright 2022 Elsevier.

Kim et al. reported a self-powered finger motion-sensing display (SMSD) based on IHN-BCP, which visualized not only finger motion via humidity but also triboelectrification, as shown in Figure 9 [111]. For self-powered properties, a triboelectric nanogenerator [112] was used. Figure 9a illustrates a schematic of the operating principle of the SMSD. As the hygroscopic quaternized poly (2-vinyl pyridine) (QP2VP) absorbed moisture, swelling of the IHN-QP2VP layer induced a structural color change, which conducted motion sensing of various finger motions, such as lateral sliding and vertical approaches. Figure 9b shows the aligned SMSD panel. Using colorimetric analysis, the SMSD panel displayed its capability as a touchless motion sensor (Figure 9d) and a visualization function without a microprocessor. However, using triboelectrification, SMSD is also capable of triboelectric sensing and visualization with image processing conducted by a microprocessor. Figure 9c shows the triboelectric voltage induced by the hand, whereas the processed visualization image clearly shows the vertical hand approach (Figure 9e).

## 9. Multi-Stimuli Sensors

The multi-stimuli sensor is an integrated sensing system that is responsive to multiple inputs, such as temperature, strain, and UV light [113]. However, integrating multiple chromic sensors in one system without interfering with individual functions is challenging, and leads to a complicated synthesis progress, yet it attracts objects for forward-looking multiple sensing applications [114]. In the aspect of chromic sensors, multi-stimuli sensors adopt materials with different chromic phenomena, such as thermochromism for temperature sensing, mechanochromism for strain sensing, photochromism for UV sensing, and electrochromism for colorimetric control.

**Figure 10:**
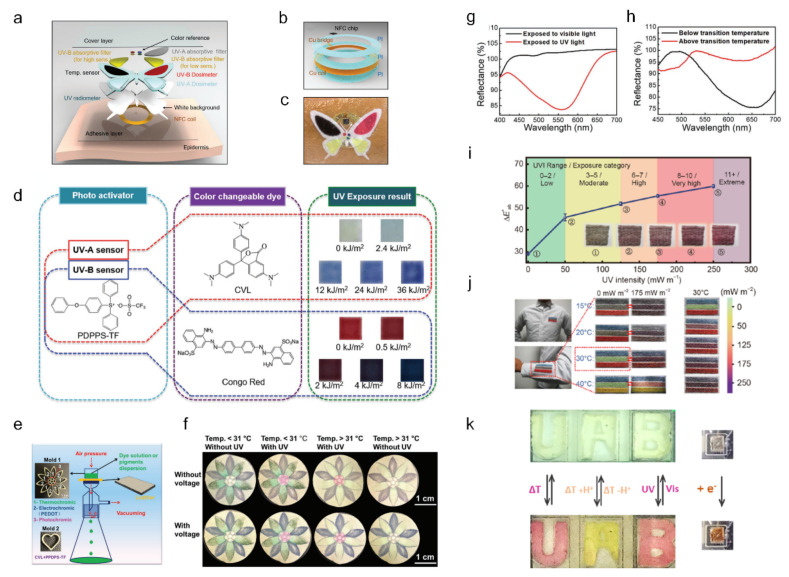
Multi-stimuli-responsive chromic device with UV exposure and temperature ((**a**–**d**) adapted with permission from ref. [113], copyright 2016 Wiley-VCH Verlag GmbH & Co., (**e**–**h**) adapted with permission from ref. [116], copyright 2021 Wiley-VCH Verlag GmbH & Co, (**i**,**j**) adapted with permission from ref. [117], copyright 2021 Springer, and (**k**) adapted with permission from ref. [120], copyright 2021 American Chemical Society). (**a**) Schematic illustrations of the various functional layers in the multi-stimuli chromic device. (**b**) Schematic illustrations of the near-field communication electronics with a temperature sensor. (**c**) Image of the fully integrated multifunctional chromic device. (**d**) The chemical response of (4-phenoxyphenyl) diphenylsulfonium triflate with crystal violet lactone and Congo red for sensing in the UV-A and UV-B bands, respectively. (**e**) Schematic and pattern of the leather-based multi-stimuli device. (**f**) Thermochromic, photochromic, electrochromic demonstration of the leather-based multi-stimuli device. (**g**) Reflectance spectra of the photochromic pigment with 365 nm UV exposure (red line) and without UV exposure (black line). (**h**) Reflectance spectra of the thermochromic pigment around the transition temperature (31 °C). (**i**) Color change of the chromic fibers depending on the UV intensities. (**j**) Photographs of a three-line striped textile showing different colors and monitoring different ambient UV indices and temperatures. (**k**) Multi-stimuli-responsive microfluidic device based on NO_2_BIPS@IG.

Araki et al. demonstrated an epidermal multi-stimuli system integrated with a temperature sensor, an UV radiometer, and UV-A and UV-B dosimeters to manage the UV solar radiation exposure to the skin [113]. Using colorimetric chemicals for each sensor, the assembled sensor was used to display the sensing state with color. Figure 10a presents schematics of the assembled multi-stimuli system, including the near-field communication system [115] (Figure 10b) for the activation of the image analysis program. By screen printing and lamination techniques, it was possible to acquire flexibility and stretchability for the skin-like epidermal patch with the entire multi-stimuli system on it, as shown in Figure 10c. For the UV dosimeter, (4-phenoxyphenyl) diphenyl sulfonium triflate was used with crystal violet lactone (UV-A dosimeter) and Congo red (UV-B dosimeter), as illustrated in Figure 10d. By analyzing the changed color of each sensing part using an associated image analysis program, this multi-stimulus system demonstrated a practical UV exposure management system.

Jia et al. developed a leather-based multi-stimuli system while preserving the full advantage of the unique micro-and nanostructures of leather with high breathability, durability, strength, and elasticity, as shown in Figure 10e–h [116]. Treating with 1-Butyl-3-methylimidazolium chloride (BMIMCl) changes the hydrophobic nature of leather so that it becomes hydrophilic, and improves conductivity, as BMIMCL forms hydrogen bonds with the leather collagen. After the BMIMCL treatment, the photochromic pigment, thermochromic pigment, and EC PEDOT: PSS were well-deposited on the leather and penetrated the leather structure, forming the leather-based UV/thermos/EC device. Figure 10e illustrates the vacuum pigmenting method and dyed pattern of each thermochromic pigment. The proposed leather-based multi-stimuli chromic device exhibited individual chromic functions, as shown in Figure 10f. The reflectance change in the visible spectra indicates significant photochromic (Figure 10g) and thermochromic behavior (Figure 10h) near a transition temperature of 31 °C.

Wei et al. reported a ‘Janus chromic’ fiber that has a dual-color mode and is responsive to UV and IR [117]. The Janus chromic fiber was designed to monitor environmental UV/IR radiation to prevent skin cancer [118,119]. A Janus chromic core with half red (photochromic dyes for UV index) and half green (thermochromic dyes for IR radiation temperature) colors was fabricated by controlling the laminar flow based on microfluidic spinning technology. With additional polylactic acid (PLA) and polyethylene glycol (PEG) layers for protection, continuous and large-scale fabrication of dual-responsive Janus chromic fibers was achieved. Figure 10i shows the UV monitoring capability of the woven chromic fiber by demonstrating the color change under a range of UV intensities of up to 11 mW m^−2^. In Figure 10j, a three-line striped textile using Janus chromic fibers is demonstrated for a visual UV/IR monitoring patch, showing various optical modulations under temperature and UV intensity. 

Santiago et al. reported a thermo-/halo-/photo-/electro- chromic device that used ion gels as a platform for mechanical flexibility, thermal stability, and chemical stability (Figure 10k) [120]. It exhibited the chromic behavior of the multi-stimuli-responsive microfluidic device under four different stimulations: temperature change, pH change, UV exposure, and electric potential. With respect to EC characteristics, the chromic device exhibited a transmittance change of 47% at 500 nm under an applied voltage of +1.2 V with a coloration time of 60.4 s.

**Figure 11:**
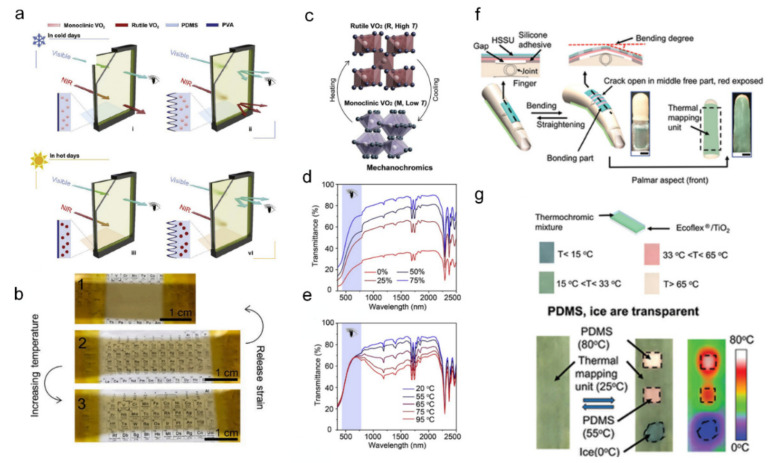
Multi-stimuli-responsive chromic device with a mechanical–thermal response ((**a**–**e**) adapted with permission from ref. [121], copyright 2020 Elsevier and (**f**,**g**) adapted with permission from ref. [124], copyright 2020 The Royal Society of Chemistry). (**a**) Schematic of four different window modes for the dispersed VO_2_ film in the PVA–PDMS bilayer structures; (**i**) normal, (**ii**) privacy, (**iii**) energy-saving, and (**vi**) simultaneous energy saving and privacy mode. (**b**) Demonstration photographs of the multi-stimuli film with (1) no strain, (2) 75 % strain, and (3) temperature of 95 °C. (**c**) Illustration of the VO_2_ phase transition between the high-temperature rutile phase and low-temperature monoclinic phase. Transmittance spectra of the dispersed VO_2_ film in the PVA–PDMS bilayer structures under privacy mode with (**d**) mechanical response and (**e**) thermal response. (**f**) Schematic and images of the wearable mechanothermal EC device on the dorsal side of a finger. (**g**) Structure and performance of the thermal mapping unit.

Ke et al. developed cephalopod-inspired mechano-thermochromic windows [121]. Through its micro-wrinkled PVA surface and well-dispersed VO_2_ nanoparticles, this multi-chromic window can control both its near-infrared transmittance and its visible spectra transmittance separately in various energy-saving/privacy situations. Figure 11a shows four different possible modes of multi-chromic windows according to thermochromic energy-saving (NIR transmittance change) and privacy mode (mechanical scattering control) controls. With respect to the mechanochromic privacy mode control, increasing the film strain induces a flattened surface, and releasing the film strain forms PVA micro-wrinkles, which leads to optical scattering for privacy. As shown in the demonstrative photograph (Figure 11b), both the thermochromic and mechanochromic functions are completely independent. The thermochromic energy-saving principle is based on the temperature-dependent reversible phase transition between the monoclinic VO_2_ and rutile VO_2_. The VO_2_ nanoparticles [122,123] block near-infrared sunlight when it is hot (rutile phase) and pass through NIR sunlight when it is cold (monoclinic phase), as illustrated in Figure 11c. The transmittance spectra (Figure 11d) show a significant decrease in visible transmittance by mechanochromic control while releasing the film strain from 75% down to 0%. Figure 11e shows the transmittance spectra at temperatures from 20 °C to 90 °C, confirming that there is no significant transmittance change in the visible range, whereas the near-infrared range shows distinct thermochromic behavior.

Zeng et al. demonstrated a wearable mechano-thermochromic device using a facile fabrication method [124]. Figure 11f shows a schematic illustration of the mechanochromic structure and thermochromic unit on the dorsal side of the finger. The mechanochromic layer consists of a vertical stacked light-shielding layer and stretchable substrate, thus the light-shielding layer ‘cracks open’ and exposes the underlying dyed layer in a stretched condition, causing colorimetric change. Figure 11g shows the thermochromic performance of the structure combined with the PDMS for thermal mapping. Upon contact with a 40 °C heat source, this multi-stimulus device showed a response time of 27 s for the color change, and was capable of detecting bending within 30% of its maximum strain.

## 10. Conclusions

This review highlights multifunctional chromic device integrated systems (various sensors, energy storage/conversion, wearable electronics, etc.) with excellent optical modulation properties, fast response, and reversible and reusable platforms. The simple redox mechanism of the chromic devices induces the color change of the chromic material due to the insertion/extraction of ions and works as a visual indicator for the external conditions (pressure, strain, biomolecules, energy, gas, ion, motion, etc.). This review focuses on advanced sensors and devices with integrated chromism, which have various advantages as practical visual detectors. It shows the specific stimuli level using optical modulation for a user-friendly system. However, some obstacles, such as various coloration states in one chromic material with good response time and thermal stability, adversely affect the information provided to the user in an unrestricted environment. In addition, for integration with various sensors and devices, mechanical flexibility and stretchability, transparent conductors, and electrochemical stability must be developed. For wearable sensors and devices used as a second skin, the most important issues are the mechanical flexibility and stretchability that they need to attach to human hands for reliable sensing operations. As a trade-off, it is possible to induce fast response with high optical modulation in nanostructured EC materials, which are not applicable for wearable sensors and devices owing to their mechanical brittleness. However, these types of nanostructured EC materials show excellent performance in energy storage, chemical sensing, and gasochromic applications. The short ion diffusion length and increased accessibility of EC materials can dramatically improve the desired performance compared to the non-structured EC materials. Mass production for applications in large-scale systems, such as electric vehicles, smart buildings, and military camouflage, should be further considered. Advanced research on integration with chromism has provided a numerous of novel operation systems with multifunctionality.

## Data Availability

Not applicable.

## References

[1] Li R., Ma X., Li J., Cao J., Gao H., Li T., Zhang X., Wang L., Zhang Q., Wang G. (2021). Flexible and high-performance elec-trochromic devices enabled by self-assembled 2D TiO_2_/MXene heterostructures. Nat. Commun..

[2] Zhong Y., Chai Z., Liang Z., Sun P., Xie W., Zhao C., Mai W. (2017). Electrochromic Asymmetric Supercapacitor Windows Enable Direct Determination of Energy Status by the Naked Eye. ACS Appl. Mater. Interfaces.

[3] Wang X., Li W., Li W., Gu C., Zheng H., Wang Y., Zhang Y.-M., Li M., Zhang S.X.-A. (2017). An RGB color-tunable turn-on electrofluorochromic device and its potential for information encryption. Chem. Commun..

[4] Xue J., Xu H., Wang S., Hao T., Yang Y., Zhang X., Song Y., Li Y., Zhao J. (2021). Design and synthesis of 2D rGO/NiO hetero-structure composites for high-performance electrochromic energy storage. Appl. Surf. Sci..

[5] Wang J., Zhang L., Yu L., Jiao Z., Xie H., Lou X.W.D., Sun W.X. (2014). A bi-functional device for self-powered electrochromic window and self-rechargeable transparent battery applications. Nat. Commun..

[6] Yun T.G., Park M., Kim D.-H., Kim D., Cheong J.Y., Bae J.G., Han S.M., Kim I.-D. (2019). All-transparent stretchable electrochromic supercapacitor wearable patch device. ACS Nano.

[7] Cai G., Wang J., Lee P.S. (2016). Next-Generation Multifunctional Electrochromic Devices. Acc. Chem. Res..

[8] Yang P., Sun P., Mai W. (2015). Electrochromic energy storage devices. Mater. Today.

[9] Wei D., Scherer M.R.J., Bower C., Andrew P., Ryhänen T., Steiner U. (2012). A Nanostructured Electrochromic Supercapacitor. Nano Lett..

[10] Arash A., Tawfik S.A., Spencer M.J., Kumar Jain S., Arash S., Mazumder A., Mayes E., Rahman F., Singh M., Bansal V. (2020). Electrically activated UV-A filters based on electrochromic MoO_3−x_. ACS Appl. Mater. Interfaces.

[11] Chaudhary A., Pathak D.K., Tanwar M., Sagdeo P.R., Kumar R. (2019). Prussian Blue-Viologen Inorganic-Organic Hybrid Blend for Improved Electrochromic Performance. ACS Appl. Electron. Mater..

[12] Zloczewska A., Celebanska A., Szot K., Tomaszewska D., Opallo M., Jönsson-Niedziolka M. (2014). Self-powered biosensor for ascorbic acid with a Prussian blue electrochromic display. Biosens. Bioelectron..

[13] Kim D., Kim J., Ko Y., Shim K., Kim J.H., You J. (2016). A facile approach for constructing conductive polymer patterns for application in electrochromic devices and flexible microelectrodes. ACS Appl. Mater. Interfaces.

[14] Shin H., Kim Y., Bhuvana T., Lee J., Yang X., Park C., Kim E. (2012). Color combination of conductive polymers for black electrochromism. ACS Appl. Mater. Interfaces.

[15] Wang L., Guo M., Zhan J., Jiao X., Chen D., Wang T. (2020). A new design of an electrochromic energy storage device with high capacity, long cycle lifetime and multicolor display. J. Mater. Chem. A.

[16] Wen R.-T., Granqvist C.G., Niklasson G.A. (2015). Eliminating degradation and uncovering ion-trapping dynamics in electrochromic WO_3_ thin films. Nat. Mater..

[17] Li J., Zhuang Y., Chen J., Li B., Wang L., Liu S., Zhao Q. (2021). Two-dimensional materials for electrochromic applications. EnergyChem.

[18] Gong S., Schwalb W., Wang Y., Chen Y., Tang Y., Si J., Shirinzadeh B., Cheng W. (2014). A wearable and highly sensitive pressure sensor with ultrathin gold nanowires. Nat. Commun..

[19] Bhatti M.R.A., Bilotti E., Zhang H., Varghese S., Verpaalen R.C.P., Schenning A.P.H.J., Bastiaansen C.W.M., Peijs T. (2020). Ultra-High Actuation Stress Polymer Actuators as Light-Driven Artificial Muscles. ACS Appl. Mater. Interfaces.

[20] Zhan Z., Lin R., Tran V.-T., An J., Wei Y., Du H., Tran T., Lu W. (2017). Paper/Carbon Nanotube-Based Wearable Pressure Sensor for Physiological Signal Acquisition and Soft Robotic Skin. ACS Appl. Mater. Interfaces.

[21] Wang C., Hwang D., Yu Z., Takei K., Park J., Chen T., Ma B., Javey A. (2013). User-interactive electronic skin for instantaneous pressure visualization. Nat. Mater..

[22] Chou H.-H., Nguyen A., Chortos A., To J.W., Lu C., Mei J., Kurosawa T., Bae W.-G., Tok J.B.-H., Bao Z. (2015). A chameleon-inspired stretchable electronic skin with interactive colour changing controlled by tactile sensing. Nat. Commun..

[23] Liu Y.-F., Liu Q., Long J.-F., Yi F.-L., Li Y.-Q., Lei X.-H., Huang P., Du B., Hu N., Fu S.-Y. (2020). Bioinspired Color-Changeable Organogel Tactile Sensor with Excellent Overall Performance. ACS Appl. Mater. Interfaces.

[24] Yu Z., Cai G., Liu X., Tang D. (2021). Pressure-Based Biosensor Integrated with a Flexible Pressure Sensor and an Electrochromic Device for Visual Detection. Anal. Chem..

[25] Liana D.D., Raguse B., Gooding J.J., Chow E. (2016). An Integrated Paper-Based Readout System and Piezoresistive Pressure Sensor for Measuring Bandage Compression. Adv. Mater. Technol..

[26] Hua Q., Sun J., Liu H., Bao R., Yu R., Zhai J., Pan C., Wang Z.L. (2018). Skin-inspired highly stretchable and conformable matrix networks for multifunctional sensing. Nat. Commun..

[27] Ershad F., Thukral A., Yue J., Comeaux P., Lu Y., Shim H., Sim K., Kim N.-I., Rao Z., Guevara R. (2020). Ultra-conformal drawn-on-skin electronics for multifunctional motion artifact-free sensing and point-of-care treatment. Nat. Commun..

[28] Larimi S.R., Nejad H.R., Oyatsi M., O’Brien A., Hoorfar M., Najjaran H. (2018). Low-cost ultra-stretchable strain sensors for monitoring human motion and bio-signals. Sens. Actuators A Phys..

[29] Ding Y., Xu T., Onyilagha O., Fong H., Zhu Z. (2019). Recent advances in flexible and wearable pressure sensors based on piezoresistive 3D monolithic conductive sponges. ACS Appl. Mater. Interfaces.

[30] Park H., Kim D.S., Hong S.Y., Kim C., Yun J.Y., Oh S.Y., Jin S.W., Jeong Y.R., Kim G.T., Ha J.S. (2017). A skin-integrated transparent and stretchable strain sensor with interactive color-changing electrochromic displays. Nanoscale.

[31] Kim D.S., Lee Y.H., Kim J.W., Lee H., Jung G., Ha J.S. (2022). A stretchable array of high-performance electrochromic devices for displaying skin-attached multi-sensor signals. Chem. Eng. J..

[32] Yu F., Liu W., Ke S.-W., Kurmoo M., Zuo J.-L., Zhang Q. (2020). Electrochromic two-dimensional covalent organic framework with a reversible dark-to-transparent switch. Nat. Commun..

[33] Panagopoulou M., Vernardou D., Koudoumas E., Tsoukalas D., Raptis Y.S. (2019). Tungsten doping effect on V2O5 thin film electrochromic performance. Electrochim. Acta.

[34] Sajitha S., Aparna U., Deb B. (2019). Ultra-Thin Manganese Dioxide-Encrusted Vanadium Pentoxide Nanowire Mats for Electrochromic Energy Storage Applications. Adv. Mater. Interfaces.

[35] Zhao S., Huang W., Guan Z., Jin B., Xiao D. (2019). A novel bis(dihydroxypropyl) viologen-based all-in-one electrochromic device with high cycling stability and coloration efficiency. Electrochim. Acta.

[36] Zhang Q., Tsai C.-Y., Li L.-J., Liaw D.-J. (2019). Colorless-to-colorful switching electrochromic polyimides with very high contrast ratio. Nat. Commun..

[37] Zheng R., Wang Y., Jia C., Wan Z., Luo J., Malik H.A., Weng X., Xie J., Deng L. (2018). Intelligent Biomimetic Chameleon Skin with Excellent Self-Healing and Electrochromic Properties. ACS Appl. Mater. Interfaces.

[38] Liu B.J.-W., Zheng J., Wang J.-L., Xu J., Li H.-H., Yu S.-H. (2013). Ultrathin W_18_O_49_ nanowire assemblies for electrochromic devices. Nano Lett..

[39] Ling H., Wu J., Su F., Tian Y., Liu Y.J. (2021). Automatic light-adjusting electrochromic device powered by perovskite solar cell. Nat. Commun..

[40] Kumar A., Williams T.L., Martin C.A., Figueroa-Navedo A.M., Deravi L.F. (2018). Xanthommatin-Based Electrochromic Displays Inspired by Nature. ACS Appl. Mater. Interfaces.

[41] Kortz C., Hein A., Ciobanu M., Walder L., Oesterschulze E. (2019). Complementary hybrid electrodes for high contrast electrochromic devices with fast response. Nat. Commun..

[42] Zhang W., Li H., Yu W.W., Elezzabi A.Y. (2020). Transparent inorganic multicolour displays enabled by zinc-based electrochromic devices. Light Sci. Appl..

[43] Shao Z., Huang A., Ming C., Bell J., Yu P., Sun Y.-Y., Jin L., Ma L., Luo H., Jin P. (2022). All-solid-state proton-based tandem structures for fast-switching electrochromic devices. Nat. Electron..

[44] Mishra S., Kumar R. (2019). Graphene nanoflakes: Foundation for improving solid state electrochemistry based electrochromic devices. Sol. Energy Mater. Sol. Cells.

[45] Lee C.H., Seok H., Jang W., Kim J.T., Park G., Kim H.-U., Rho J., Kim T., Chung T.D. (2021). Bioaerosol monitoring by integrating DC impedance microfluidic cytometer with wet-cyclone air sampler. Biosens. Bioelectron..

[46] Kim H.U., Koyappayil A., Seok H., Aydin K., Kim C., Park K.Y., Jeon N., Kang W.S., Lee M.H., Kim T. (2021). Concurrent and Selective Determination of Dopamine and Serotonin with Flexible WS_2_/Graphene/Polyimide Electrode Using Cold Plasma. Small.

[47] Kim H.-U., Kim H.Y., Seok H., Kanade V., Yoo H., Park K.-Y., Lee J.-H., Lee M.-H., Kim T. (2020). Flexible MoS_2_–Polyimide Electrode for Electrochemical Biosensors and Their Applications for the Highly Sensitive Quantification of Endocrine Hormones: PTH, T3, and T4. Anal. Chem..

[48] Capoferri D., Álvarez-Diduk R., Del Carlo M., Compagnone D., Merkoçi A. (2018). Electrochromic Molecular Imprinting Sensor for Visual and Smartphone-Based Detections. Anal. Chem..

[49] Yeon S.Y., Seo M., Kim Y., Hong H., Chung T.D. (2022). Paper-based electrochromic glucose sensor with polyaniline on indium tin oxide nanoparticle layer as the optical readout. Biosens. Bioelectron..

[50] Sun X., Zhang H., Hao S., Zhai J., Dong S. (2019). A Self-Powered Biosensor with a Flake Electrochromic Display for Electrochemical and Colorimetric Formaldehyde Detection. ACS Sens..

[51] Davy N.C., Sezen-Edmonds M., Gao J., Lin X., Liu A., Yao N., Kahn A., Loo Y.-L. (2017). Pairing of near-ultraviolet solar cells with electrochromic windows for smart management of the solar spectrum. Nat. Energy.

[52] Laschuk N.O., Ebralidze I.I., Easton E.B., Zenkina O.V. (2021). Systematic Design of Electrochromic Energy Storage Devices Based on Metal-Organic Monolayers. ACS Appl. Energy Mater..

[53] Wang J.-L., Sheng S.-Z., He Z., Wang R., Pan Z., Zhao H.-Y., Liu J.-W., Yu S.-H. (2021). Self-Powered Flexible Electrochromic Smart Window. Nano Lett..

[54] Cossari P., Pugliese M., Gambino S., Cannavale A., Maiorano V., Gigli G., Mazzeo M. (2018). Fully integrated electrochromic-OLED devices for highly transparent smart glasses. J. Mater. Chem. C.

[55] He Z., Gao B., Li T., Liao J., Liu B., Liu X., Wang C., Feng Z., Gu Z. (2018). Piezoelectric-driven self-powered patterned electrochromic supercapacitor for human motion energy harvesting. ACS Sustain. Chem. Eng..

[56] Stec G.J., Lauchner A., Cui Y., Nordlander P., Halas N.J. (2017). Multicolor Electrochromic Devices Based on Molecular Plasmonics. ACS Nano.

[57] Li X., Du Z., Song Z., Li B., Wu L., Liu Q., Zhang H., Li W. (2018). Bringing Hetero-Polyacid-Based Underwater Adhesive as Printable Cathode Coating for Self-Powered Electrochromic Aqueous Batteries. Adv. Funct. Mater..

[58] An T., Ling Y., Gong S., Zhu B., Zhao Y., Dong D., Yap L.W., Wang Y., Cheng W. (2019). A wearable second skin-like multifunctional supercapacitor with vertical gold nanowires and electrochromic polyaniline. Adv. Mater. Technol..

[59] Kim S.Y., Jang Y.J., Kim Y.M., Lee J.K., Moon H.C. (2022). Tailoring Diffusion Dynamics in Energy Storage Ionic Conductors for High-Performance, Multi-Function, Single-Layer Electrochromic Supercapacitors. Adv. Funct. Mater..

[60] Edwards P.P., Kuznetsov V.L., David W.I.F., Brandon N.P. (2008). Hydrogen and fuel cells: Towards a sustainable energy future. Energy Policy.

[61] Keshri S., Kumar A., Kabiraj D. (2012). Tailoring of optical and gas sensitivity behaviors of WO^3^ films by low energy Ar+ ion implantation. Thin Solid Films.

[62] Lee Y.-A., Kalanur S.S., Shim G., Park J., Seo H. (2017). Highly sensitive gasochromic H_2_ sensing by nano-columnar WO_3_-Pd films with surface moisture. Sens. Actuators B Chem..

[63] Zhou B., Feng W., Gao G., Wu G., Chen Y., Li W. (2017). A low cost preparation of WO_3_ nanospheres film with improved thermal stability of gasochromic and its application in smart windows. Mater. Res. Express.

[64] Gao G., Xue S., Wang H., Zhang Z., Shen J., Wu G. (2022). Medium-scale production of gasochromic windows by sol-gel. J. Sol-Gel Sci. Technol..

[65] Gao G., Xue S., Wang H., Zhang Z., Wu G., Debela T.T., Kang H.S. (2021). Highly Thermally Stable and Transparent WO_3_–SiO_2_ Gasochromic Films Obtained by an Automated Printing Method. ACS Sustain. Chem. Eng..

[66] Favier F., Walter E.C., Zach M.P., Benter T., Penner R.M. (2001). Hydrogen Sensors and Switches from Electrodeposited Palladium Mesowire Arrays. Science.

[67] Yang F., Taggart D.K., Penner R.M. (2009). Fast, Sensitive Hydrogen Gas Detection Using Single Palladium Nanowires that Resist Fracture. Nano Lett..

[68] Zeng X.Q., Latimer M.L., Xiao Z.L., Panuganti S., Welp U., Kwok W.K., Xu T. (2011). Hydrogen Gas Sensing with Networks of Ultrasmall Palladium Nanowires Formed on Filtration Membranes. Nano Lett..

[69] Boudiba A., Roussel P., Zhang C., Olivier M.-G., Snyders R., Debliquy M. (2013). Sensing mechanism of hydrogen sensors based on palladium-loaded tungsten oxide (Pd–WO_3_). Sens. Actuators B Chem..

[70] Kolmakov A., Klenov D.O., Lilach Y., Stemmer A.S., Moskovits M. (2005). Enhanced Gas Sensing by Individual SnO_2_ Nanowires and Nanobelts Functionalized with Pd Catalyst Particles. Nano Lett..

[71] Khoobiar S.J.T. (1964). Particle to Particle Migration of Hydrogen Atoms on Platinum-Alumina Catalysts from Particle to Neighboring Particles. J. Phys. Chem..

[72] Marcinkowski M.D., Jewell A.D., Stamatakis M., Boucher M.B., Lewis E.A., Murphy C., Kyriakou G., Sykes E.C.H. (2013). Controlling a spillover pathway with the molecular cork effect. Nat. Mater..

[73] Behbahani M.A., Ranjbar M., Kameli P., Salamati H. (2013). Hydrogen sensing by wet-gasochromic coloring of PdCl_2_ (*aq*)/WO_3_ and the role of hydrophilicity of tungsten oxide films. Sens. Actuators B Chem..

[74] Foroushani F.T., Tavanai H., Ranjbar M., Bahrami H. (2018). Fabrication of tungsten oxide nanofibers via electrospinning for gasochromic hydrogen detection. Sens. Actuators B Chem..

[75] Nikfarjam A., Fardindoost S., Zad A.I. (2013). Fabrication of Pd Doped WO_3_ Nanofiber as Hydrogen Sensor. Polymers.

[76] Liu B., Cai D., Liu Y., Wang D., Wang L., Wang Y., Li H., Li Q., Wang T. (2014). Improved room-temperature hydrogen sensing performance of directly formed Pd/WO_3_ nanocomposite. Sens. Actuators B Chem..

[77] Kalanur S.S., Yoo I.-H., Seo H. (2017). Pd on MoO_3_ nanoplates as small-polaron-resonant eye-readable gasochromic and electrical hydrogen sensor. Sens. Actuators B Chem..

[78] Alsaif M.M., Latham K., Field M.R., Yao D.D., Medehkar N.V., Beane G.A., Kaner R.B., Russo S.P., Ou J.Z., Kalantar-zadeh K. (2014). Tunable plasmon resonances in two-dimensional molybdenum oxide nanoflakes. Adv. Mater..

[79] He T., Yao J. (2007). Photochromic materials based on tungsten oxide. J. Mater. Chem..

[80] Tahini H.A., Tan X., Lou S.N., Scott J., Amal R., Ng Y.H., Smith S.C. (2016). Mobile polaronic states in α-MoO_3_: An ab initio investigation of the role of oxygen vacancies and alkali ions. ACS Appl. Mater. Interfaces.

[81] You K., Cao F., Wu G., Zhao P., Huang H., Wang Z., Hu Y., Gu H., Wang J. (2019). Room-temperature H_2_ gasochromic behavior of Pd-modified MoO_3_ nanowire labels. Mater. Chem. Phys..

[82] Saenger M.F., Höing T., Robertson B.W., Billa R.B., Hofmann T., Schubert E., Schubert M. (2008). Polaron and phonon properties in proton intercalated amorphous tungsten oxide thin films. Phys. Rev. B.

[83] Han S.-I., Lee S.Y., Duy L.T., Seo H. (2021). Reversible gasochromic hydrogen sensing of mixed-phase MoO_3_ with multi-layered Pt/Ni/Pt catalyst. Int. J. Hydrogen Energy.

[84] Yao D.D., Ou J.Z., Latham K., Zhuiykov S., O’Mullane A.P., Kalantar-Zadeh K. (2012). Electrodeposited α-and β-phase MoO_3_ films and investigation of their gasochromic properties. Cryst. Growth Des..

[85] Nie L., Guo X., Gao C., Wu X., Chen J., Peng L. (2022). Optical H_2_-sensing properties of ordered porous WO_3_ films prepared by colloidal template method. J. Mater. Sci. Mater. Electron..

[86] Xue S., Gao G., Zhang Z., Jiang X., Shen J., Wu G., Dai H., Xu Y., Xiao Y. (2021). Nanoporous WO_3_ Gasochromic Films for Gas Sensing. ACS Appl. Nano Mater..

[87] Chen M., Zou L., Zhang Z., Shen J., Li D., Zong Q., Gao G., Wu G., Zhang Z. (2018). Tandem gasochromic-Pd-WO_3_/graphene/Si device for room-temperature high-performance optoelectronic hydrogen sensors. Carbon.

[88] Feng W., Wu G., Gao G. (2014). Ordered mesoporous WO_3_ film with outstanding gasochromic properties. J. Mater. Chem. A.

[89] Qi W., Gao G., Wu G., Wang H. (2019). Flexible gasochromic films with favorable high temperature resistance and energy efficiency. Sol. Energy Mater. Sol. Cells.

[90] Wang H., Gao G., Wu G., Zhao H., Qi W., Chen K., Zhang W., Li Y. (2019). Fast hydrogen diffusion induced by hydrogen pre-split for gasochromic based optical hydrogen sensors. Int. J. Hydrogen Energy.

[91] Hu C.-W., Yamada Y., Yoshimura K., Takahashi A., Watanabe H., Tajima K., Kawamoto T. (2018). High contrast gasochromism of wet processable thin film with chromic and catalytic nanoparticles. J. Mater. Chem. C.

[92] Hu C.-W., Yamada Y., Yoshimura K. (2016). Fabrication of nickel oxyhydroxide/palladium (NiOOH/Pd) thin films for gasochromic application. J. Mater. Chem. C.

[93] Lu Y.-R., Hsu H.-H., Chen J.-L., Chang H.-W., Chen C.-L., Chou W.-C., Dong C.-L. (2016). Atomic and electronic aspects of the coloration mechanism of gasochromic Pt/Mo-modified V_2_O_5_ smart films: An in situ X-ray spectroscopic study. Phys. Chem. Chem. Phys..

[94] Ho Y., Chang C., Wei D., Dong C., Chen C., Chen J., Jang W., Hsu C., Chan T., Kumar K. (2013). Characterization of gasochromic vanadium oxides films by X-ray absorption spectroscopy. Thin Solid Films.

[95] Chen C.L., Dong C.L., Ho Y.K., Chang C.C., Wei D.H., Chan T.C., Chen J.L., Jang W.L., Hsu C.C., Kumar K. (2013). Electronic and atomic structures of gasochromic V_2_O_5_ films. Europhys. Lett..

[96] Chen J.-L., Chang C.-C., Ho Y.-K., Chen C.L., Hsu C.-C., Jang W.-L., Wei D.-H., Dong C.-L., Pao C.-W., Lee J.-F. (2015). Behind the color switching in gasochromic VO_2_. Phys. Chem. Chem. Phys..

[97] Liu Y., Chen J., Peng L., Deng N., Ding W.J.I. (2019). Fabrication and optical property improvement of gasochromic switchable mirror based on Pd/MgNb_2_O_5_ thin film. Int. J. Hydrogen Energy.

[98] Hu C.-W., Yamada Y., Yoshimura K. (2018). Poly (3,4-alkylenedioxythiophenes): PXDOTs electrochromic polymers as gasochromic materials. Sol. Energy Mater. Sol. Cells.

[99] Chang L.W., Magos L., Suzuki T. (1996). Toxicology of Metal.

[100] Ye B.C., Yin B.C. (2008). Highly sensitive detection of mercury (II) ions by fluorescence polarization enhanced by gold nanoparticles. Angew. Chem..

[101] Lee J.S., Han M.S., Mirkin C.A. (2007). Colorimetric detection of mercuric ion (Hg^2+^) in aqueous media using DNA-functionalized gold nanoparticles. Angew. Chem. Int. Ed..

[102] Si Y., Wang X., Li Y., Chen K., Wang J., Yu J., Wang H., Ding B. (2014). Optimized colorimetric sensor strip for mercury(ii) assay using hierarchical nanostructured conjugated polymers. J. Mater. Chem. A.

[103] Price C., Carroll J., Clare T.L. (2018). Chemoresistive and photonic hydrogel sensors of transition metal ions via Hofmeister series principles. Sens. Actuators B Chem..

[104] de Tacconi N.R., Rajeshwar K., Lezna R.O. (2003). Metal hexacyanoferrates: Electrosynthesis, in situ characterization, and applications. Chem. Mater..

[105] Yao T., Tu Q., Han X., Zhang L., Wang D.-E., Li M., Chen S., Wang J. (2014). SiO_2_ nanoparticles and diphenylcarbazide doped polymethylmethacrylate electrospun fibrous film for Cd^2+^ colorimetric detection. Anal. Methods.

[106] Ding B., Si Y., Wang X., Yu J., Feng L., Sun G. (2011). Label-free ultrasensitive colorimetric detection of copper (II) ions utilizing polyaniline/polyamide-6 nano-fiber/net sensor strips. J. Mater. Chem..

[107] Kim E.H., Cho S.H., Lee J.H., Jeong B., Kim R.H., Yu S., Lee T.-W., Shim W., Park C. (2017). Organic light emitting board for dynamic interactive display. Nat. Commun..

[108] Lee S.W., Cho S.H., Kang H.S., Kim G., Kim J.S., Jeong B., Kim E.H., Yu S., Hwang I., Han H. (2018). Electroluminescent pressure-sensing displays. ACS Appl. Mater. Interfaces.

[109] Lee S.W., Baek S., Park S.-W., Koo M., Kim E.H., Lee S., Jin W., Kang H., Park C., Kim G. (2020). 3D motion tracking display enabled by magneto-interactive electroluminescence. Nat. Commun..

[110] Welch V.L., Vigneron J.-P. (2007). Beyond butterflies—The diversity of biological photonic crystals. Opt. Quantum Electron..

[111] Kim T., Lee J.W., Park C., Lee K., Lee C.E., Lee S., Kim Y., Kim S., Jeon S., Ryu D.Y. (2022). Self-powered finger motion-sensing structural color display enabled by block copolymer photonic crystal. Nano Energy.

[112] Ma J., Zhu J., Ma P., Jie Y., Wang Z.L., Cao X. (2020). Fish Bladder Film-Based Triboelectric Nanogenerator for Noncontact Position Monitoring. ACS Energy Lett..

[113] Araki H., Kim J., Zhang S., Banks A., Crawford K.E., Sheng X., Gutruf P., Shi Y., Pielak R.M., Rogers J.A. (2017). Materials and device designs for an epidermal UV colorimetric dosimeter with near field communication capabilities. Adv. Funct. Mater..

[114] Kim J.J., Wang Y., Wang H., Lee S., Yokota T., Someya T. (2021). Skin Electronics: Next-Generation Device Platform for Virtual and Augmented Reality. Adv. Funct. Mater..

[115] Kim J., Banks A., Cheng H., Xie Z., Xu S., Jang K.-I., Lee J.W., Liu Z., Gutruf P., Huang X. (2015). Epidermal Electronics with Advanced Capabilities in Near-Field Communication. Small.

[116] Jia L., Zeng S., Ding H., Smith A.T., LaChance A.M., Farooqui M.M., Gao D., Ma J., Sun L. (2021). Leather-Based Multi-Stimuli Responsive Chromisms. Adv. Funct. Mater..

[117] Wei Y., Zhang W., Hou C., Zhang Q., Li Y., Wang H. (2021). Independent dual-responsive Janus chromic fibers. Sci. China Mater..

[118] Dong K., Goyarts E., Rella A., Pelle E., Wong Y.H., Pernodet N. (2020). Age Associated Decrease of MT-1 Melatonin Receptor in Human Dermal Skin Fibroblasts Impairs Protection against UV-Induced DNA Damage. Int. J. Mol. Sci..

[119] Sklar L.R., Almutawa F., Lim H.W., Hamzavi I. (2012). Effects of ultraviolet radiation, visible light, and infrared radiation on erythema and pigmentation: A review. Photochem. Photobiol. Sci..

[120] Santiago S., Giménez-Gómez P., Muñoz-Berbel X., Hernando J., Guirado G. (2021). Solid Multiresponsive Materials Based on Nitrospiropyran-Doped Ionogels. ACS Appl. Mater. Interfaces.

[121] Ke Y., Zhang Q., Wang T., Wang S., Li N., Lin G., Liu X., Dai Z., Yan J., Yin J. (2020). Cephalopod-inspired versatile design based on plasmonic VO2 nanoparticle for energy-efficient mechano-thermochromic windows. Nano Energy.

[122] Budai J.D., Hong J., Manley M.E., Specht E.D., Li C.W., Tischler J.Z., Abernathy D.L., Said A.H., Leu B.M., Boatner L. (2014). Metallization of vanadium dioxide driven by large phonon entropy. Nature.

[123] Liu M., Su B., Kaneti Y.V., Chen Z., Tang Y., Yuan Y., Gao Y., Jiang L., Jiang X., Yu A. (2017). Dual-Phase Transformation: Spontaneous Self-Template Surface-Patterning Strategy for Ultra-transparent VO_2_ Solar Modulating Coatings. ACS Nano.

[124] Zeng S., Sun H., Park C., Zhang M., Zhu M., Yan M., Chov N., Li E., Smith A.T., Xu G. (2020). Multi-stimuli responsive chromism with tailorable mechanochromic sensitivity for versatile interactive sensing under ambient conditions. Mater. Horiz..

